# Constructing founder sets under allelic and non-allelic homologous recombination

**DOI:** 10.1186/s13015-023-00241-3

**Published:** 2023-09-29

**Authors:** Konstantinn Bonnet, Tobias Marschall, Daniel Doerr

**Affiliations:** Institute for Medical Biometry and Bioinformatics, Medical Faculty, and Center for Digital Medicine, Heinrich Heine University, Moorenstr. 5, 40225 Düsseldorf, Germany

**Keywords:** Founder set reconstruction, Variation graph, Pangenomics, NAHR, Homologous recombination

## Abstract

Homologous recombination between the maternal and paternal copies of a chromosome is a key mechanism for human inheritance and shapes population genetic properties of our species. However, a similar mechanism can also act between different copies of the same sequence, then called *non-allelic homologous recombination (NAHR)*. This process can result in genomic rearrangements—including deletion, duplication, and inversion—and is underlying many genomic disorders. Despite its importance for genome evolution and disease, there is a lack of computational models to study genomic loci prone to NAHR. In this work, we propose such a computational model, providing a unified framework for both (allelic) homologous recombination and NAHR. Our model represents a set of genomes as a graph, where haplotypes correspond to walks through this graph. We formulate two founder set problems under our recombination model, provide flow-based algorithms for their solution, describe exact methods to characterize the number of recombinations, and demonstrate scalability to problem instances arising in practice.

**Supplementary Information:**

The online version contains supplementary material available at 10.1186/s13015-023-00241-3.

## Background

Twenty years ago, Esko Ukkonen introduced the problem of inferring founder sets from haplotyped single nucleotide polymorphism (SNP) sequences under allelic recombination [1]. Ukkonen’s work has since inspired a wealth of research addressing various aspects and applications of founder set reconstruction ranging from the reconstruction of ancestral recombinations and pangenomics to applications in phage evolution [2–4]. In its original setting, the problem sets out from a given set of *m* sequences of equal length *n*, where characters across sequences residing at the same index position correspond to a SNP. It then asks for a smallest set of sequences, called *founder set*, such that each given sequence can be constructed through a series of crossovers between sequences of the founder set, where each segment between two successive recombinations must meet a minimum length threshold. The *Minimum Founder Set* problem is NP-complete in general [5], but is solvable in linear time for the special case of founder sets of size two [1, 6]. Since its introduction, various heuristics and approximations have been proposed [6–8]. A variant of this problem restricts crossovers to coincide at certain positions, thereby decomposing the input sequences into a universally shared succession of blocks. The resulting problem, known as *Minimum Segmentation Problem* is polynomial [9]. In his seminal paper, Ukkonen devised a $O(n^2m)$ algorithm for its solution which has been substantially improved by Norri et al. [10] to linear time, i.e. *O*(*nm*) by exploiting the positional Burrows-Wheeler transform [11].

Just like the Minimum Founder Set Problem, the vast majority of population genetic analyses and genome-wide association studies have been focused on SNPs in the past decades, neglecting more complex forms of variation—mostly for technical difficulties in detecting them. In particular, structural variants (SVs), commonly defined as variants of at least 50bp, have posed substantial challenges and studies based on short sequencing reads typically detect less than half of all SVs present in a genome [12]. Recent technological and algorithmic advances help to overcome these limitations [13]. Long read technologies now enable haplotype-resolved *de novo* assembly of human genomes [14], which in turn enables a much more complete ascertainment of SVs [15]. In 2022, the first complete telomere-to-telomere assembly of a human genome was announced [16], heralding a new era of genomics where high-quality, haplotype-resolved assemblies of complex repetitive genomic structures become broadly available. Presently, the Human Pangenome Reference Consortium (HPRC), is applying these techniques to generate a large panel of haplotype-resolved genome assemblies from samples of diverse ancestries [17, 18]. These emerging data sets enable studying genetic loci involving duplicated sequence, called *segmental duplications (SDs)*, which are amenable to NAHR, are therefore highly mutable, and show complicated evolutionary trajectories [19, 20]. The T2T-CHM13 study alone reports over 40 thousand segmental duplications that amount to 202Mb ($6.6\%$ of the human genome) [16].

**Fig. 1:**
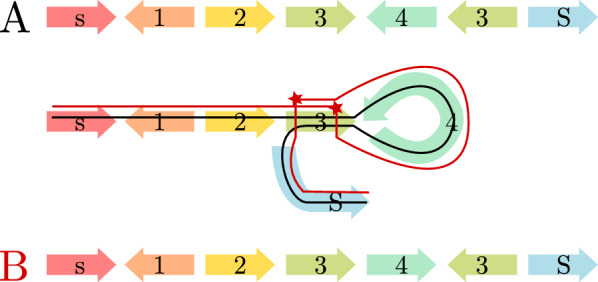
*Illustration of an NAHR-mediated inversion.* Haplotype **A** (black line) represents the original configuration, while haplotype **B** (red line) can be derived from *A* by two recombination events between inverted repeats of genomic marker 3 as indicated by the red stars

Interestingly, at loci with highly similar segments arranged in opposite orientations, such as Segment 3 in Fig. 1, NAHR can lead to *inversion*, i.e. the reversal of the interior sequence (Segment 4 in Fig. 1). Because of being flanked by a pair of copies of the same sequence (cf. Segment 3) that often comprises tens of thousands of bases, such events have been largely undetectable by sequencing technologies with read lengths below the length of the duplicated sequence; in particular by conventional short read sequencing. Recent studies applying multiple technologies reveal that inversions affect tens of megabases of sequence in a typical human genome [21]. Unlike most other classes of genetic variation, inversions are often *recurrent* with high mutation rates, that is, the same events have happened multiple times in human history [22]. Depending on the structures of duplicated sequence at a particular locus, individual human haplotypes can differ in their potential for NAHR. This can have important implications for the risk for a range of genetic disorders caused by NAHR-mediated mutations [22].

In the past two decades, various mathematical models and algorithms to study genome rearrangements have been proposed. These range from the classic reversal [23, 24] and transposition [25] model to composed models for two or more balanced rearrangements [26, 27], to generalized models such as the popular *Double Cut and Join (DCJ)* [28, 29]. As the research in this field continues, advanced models can additionally accommodate one or more types of unbalanced rearrangements, i.e., deletion, insertion, and duplication [30, 31]. Yet, none of these models adequately considers sequence similarity as a prerequisite for NAHR, which is a key molecular mechanism shaping many complex loci in the human genome. In summary, there are now technological opportunities to study the population history of recalcitrant SD loci that are prone to genome rearrangements and relevant to disease, but computational models to facilitate this have so far been lacking.

This work addresses this deficit by proposing a rearrangement model that is based on the molecular mechanism of homologous recombination and by solving variants of Ukkonen’s Founder Set Problem that can provide insights into the evolution of complex loci driven by NAHR. The genome model underlying the approach at hand represents DNA sequences at a level of abstraction where they are already decomposed into genomic markers with assigned homologies. Here, our notion of homology is a synonym for *high DNA sequence similarity*, as we adopt the terminology underlying the concept of homologous recombination. Our model permits recombination events to occur between homologous markers independent of their position within or between haplotypes, as long as the markers’ orientations are respected. In other words, a marker can only recombine with a homologous marker alongside the same direction, as illustrated by Fig. 1, because a recombination event can only occur between homologous markers if they are aligned to each other. By virtue of recapitulating the underlying NAHR, it implicitly allows for all the rearrangements this molecular mechanism can give rise to, including deletion, duplication, and inversion.

Marker decomposition and homology assignment can be done in practice with genome graph building tools such as MBG [32], minigraph [33], or pggb [34]. Our algorithms can work with any *variation graph* or *pangenome graph* with nodes corresponding to homologous DNA segments and edges between segments corresponding to observed adjacencies in a given set of haplotypes.

## Methods

### Preliminaries

A *(genomic) marker* *m* is an element of the finite universe of markers denoted by ${\mathcal {M}}$, and is associated with a fragment of a double-stranded DNA molecule. Each marker can be traversed in *forward* and *reverse* direction. A marker in forward orientation (which is the default orientation) is traversed from left to right. Overline notation $\overline{m}$ indicates the reversal of a marker *m*, which is carried out relative to its orientation, i.e., $\overline{\overline{m}} = m$. Similarly, $\overline{{\mathcal {M}}}$ represents the set of all reversed markers. We designate two forward markers $\{s, S\} \subseteq {\mathcal {M}}$ as *terminal markers*. In what follows, we study *terminal sequences*, that is, sequences drawn from the alphabet of oriented markers ${\mathcal {M}}\cup \overline{{\mathcal {M}}}$ that start with *s* or $\overline{S}$, end in *S* or $\overline{s}$ and do not contain any further terminal markers in between. A terminal sequence can be traversed in forward and reverse direction. A *haplotype* is a terminal sequence that starts with *s* (*source*) and ends with *S* (*sink*).

#### Example 1

Consider in the following two sequences of genomic markers *A* and *X* drawn from the universe of markers ${\mathcal {M}} = \{\texttt {s}, \texttt {1}, \texttt {2}, \texttt {3}, \texttt {4}, \texttt {S}\}$, where $A = \texttt {s}\overline{\texttt {1}}{} \texttt {23}\overline{\texttt {4}}\overline{\texttt {3}}{} \texttt {S}$ and $X = \texttt {s}\overline{\texttt {1}}{} \texttt {234}\overline{\texttt {3}}\overline{\texttt {2}}{} \texttt {1}\overline{\texttt {s}}$. Sequence *A* starts and ends with terminal markers $\texttt {s}$ and $\texttt {S}$, respectively, thus constituting a *haplotype* over ${\mathcal {M}}$. Conversely, *X* starts with $\texttt {s}$ and ends in $\overline{\texttt {s}}$ and therefore is a terminal sequence, but not a *haplotype*.

Given a sequence *A*, |*A*| indicates the length of *A* which corresponds to the number of *A*’s constituting elements. $\overline{A}$ defines the *reverse complementation* of sequence *A*, i.e., the simultaneous reversal of the sequence and its constituting elements. The element at the *i*th position in sequence *A* is denoted by *A*[*i*]. A *segment* of sequence *A* starting at position *i* and ending at and including position *j* is denoted by *A*[*i*..*j*]. Then, $A[..i]:= A[1..i]$ and $A[i..]:= A[i..|A|]$ denote the *prefix* and *suffix* of *A*, respectively. Given two sequences *A* and *B*, then $B \lhd A$ indicates that *B* is a segment of *A*, i.e., $|B| \le |A|$ and there exists some $i \in [1, |A|-|B|]$ with $B = A[i..i+|B|-1]$. Finally, the operator “$+$” indicates the concatenation of two sequences.

#### Example 1

(cont’d) The length of *A* is $|A| = 7$; its reverse complement is $\overline{A} = {\overline{\texttt {S}}{} \texttt {34}\overline{\texttt {3}}\overline{\texttt {2}}{} \texttt {1}\overline{\texttt {s}}}$; *A*[4..6] is a segment of *A* and corresponds to sequence ${\texttt {3}\overline{\texttt {4}}\overline{\texttt {3}}}$, and consequently ${\texttt {3}\overline{\texttt {4}}\overline{\texttt {3}}} \subseteq A$ holds true; The segments $X[..6] = {\texttt {s}\overline{\texttt {1}}{} \texttt {234}\overline{\texttt {3}}}$ and $A[7..] = \texttt {S}$ are a prefix and a suffix of *X* and *A*, respectively; The concatenation of prefix *X*[..6] and suffix *A*[7..] results in haplotype $X[..6] + A[7..] = {\texttt {s}\overline{\texttt {1}}{} \texttt {234}\overline{\texttt {3}}{} \texttt {S}}$.

A *recombination* is an operation that acts on a shared oriented marker *m* of any two terminal sequences *A* and *B*: let $A[i] = B[j] = m$; recombination $\chi (A, B, i, j)$ produces terminal sequence $C = A[..i] + B[j+1..]$. For a given set of haplotypes ${\mathcal {H}}$, ${{\,\textrm{span}\,}}({\mathcal {H}})$ denotes the *span*, i.e., the set of all *haplotypes* generated by applying $\chi$ on haplotypes ${\mathcal {H}}$ and the resulting terminal sequences. More precisely, let $\Theta$ be the universe of terminal sequences, defined recursively by ${\mathcal {H}}\cup \overline{{\mathcal {H}}}\subseteq \Theta$ such that for any $A, B \in \Theta$ with some $A[i] = B[j]$ the recombinant $C = A[..i] + B[j+1..]$ and its reverse complement ${{\overline{C}}}$ is also in $\Theta$. Then ${{\,\textrm{span}\,}}({\mathcal {H}})$ $:= \{A \in \Theta \mid A \text { is a haplotype}\}$. Accordingly, we also say that “${\mathcal {H}}$ is a *generating set* of ${{\,\textrm{span}\,}}({\mathcal {H}}$)”. Conversely, given any (possibly infinite) set of haplotypes ${\mathcal {S}}$ and some ${\mathcal {H}}\subseteq {\mathcal {S}}$, ${\mathcal {H}}$ is a generating set of ${\mathcal {S}}$ if and only if ${{\,\textrm{span}\,}}({\mathcal {H}}) = {\mathcal {S}}$.

#### Example 1

(cont’d) Recombination $\chi (A, \overline{A}, 4, 2)$ produces terminal sequence $X = {\texttt {s}\overline{\texttt {1}}{} \texttt {234}\overline{\texttt {3}}\overline{\texttt {2}}{} \texttt {1}\overline{\texttt {s}}}$. Subsequent recombination $\chi (X, A, 6, 6)$ produces haplotype $B = {\texttt {s}\overline{\texttt {1}}{} \texttt {234}\overline{\texttt {3}}{} \texttt {S}}$. If $\{A\}$ is a given set of haplotypes, then ${{\,\textrm{span}\,}}(\{A\}) = \{A, B\}$.

In this paper, we study the following three problems:

#### Problem 1

(Founder Set) Given a set of haplotypes ${\mathcal {H}}$, find a generating set ${\mathcal {F}}$ such that ${{\,\textrm{span}\,}}({\mathcal {F}}) = {{\,\textrm{span}\,}}({\mathcal {H}})$ and $\sum _{A \in {\mathcal {F}}} |A|$ is minimized.

We minimize total length because current knowledge on the evolution of complex genomic loci indicates their contained segmental duplications often causes them to expand over time [35]. Consequently we expect ancestral loci to be more compact and contain fewer duplications. We prefer this formulation over minimizing the founder set’s cardinality, because the latter would allow for solutions with founder sequences of unbounded length, which is biologically irrelevant. We call a solution to Problem 1 a *founder set* and its members *founder sequences*. The following problem is related to Ukkonen’s Minimum Segmentation Problem [1]:

#### Problem 2

(Recombination Count) Given a set of terminal sequences ${\mathcal {T}}$ and a terminal sequence *Q*, count the number of recombinations *r* of the form $A_{k+1} = \chi (A_k, T_k, \cdot , \cdot )$, with $0 \le k \le r$ and $T_0,..,T_r \in {\mathcal {T}}$, that are necessary to generate *Q* from ${\mathcal {T}}$, i.e., $A_0 \in {\mathcal {T}}$ and $Q=A_r$, if feasible and report its infeasibility otherwise.

At last, the combination of Problems 1 and 2 motivates the following:

#### Problem 3

(Parsimonious Founder Set) Given a set of haplotypes ${\mathcal {H}}$, find a founder set ${\mathcal {F}}$ that minimizes the total number of recombinations to generate all founder sequences from ${\mathcal {H}}$.

### Constructing founder sets

In this section, we present a three-step solution to Problem 1 that is based on a network flow analysis of the *variation graph* over the input set of haplotypes. To this end, we introduce the notion of variation graphs and describe their construction for our specific setting. Subsequently, we define network flow and detail how a founder set can be derived. Our proposed network flow problem is subordinate to the Chinese Postman Problem on edge-colored multigraphs for which Gutin et al. proposed a polynomial algorithm [36]. Consequently, all other steps of our solution being polynomial, Problem 1 can be solved in polynomial time. However, we propose an integer linear program in lieu of Gutin et al’s impractical algorithm. Then, in Section Results we show feasibility of our approach in experiments on simulated variation graphs and an exemplar biological data set.

*Variation graph construction.* We now address the construction of variation graph $G_{\mathcal {H}}= (V, E\cup \overrightarrow{E})$ from a given set of haplotypes ${\mathcal {H}}$. Graph $G_{\mathcal {H}}$ is an undirected edge-colored multigraph where each edge can have one of two colors corresponding to their membership in edge sets *E* and $\overrightarrow{E}$. In constructing $G_{\mathcal {H}}$, each marker *m* of the universe of forward-oriented markers ${\mathcal {M}}$ is represented by a tuple of its *extremities* $(m^\text {t}, m^\text {h})$ also called “*tail*” and “*head*” of *m*, respectively, and its reverse-oriented counterpart $\overline{m}$ is represented as $(m^\text {h}, m^\text {t})$. (Note that our notation is based on common practice of illustrating markers as arrows, that, in natural reading direction, face from left, i.e., tail of the arrow, to right, i.e., head of the arrow.) Node set *V* of graph $G_{\mathcal {H}}$ corresponds to the set of all marker extremities, and each marker $m \in {\mathcal {M}}$ gives rise to one *marker edge* $\{m^\text {t}, m^\text {h}\} \in \overrightarrow{E}$. Further, any two (not necessarily distinct) nodes $m_1^b, m_2^c \in V$ are connected by one *adjacency edge* $\{m_1^b, m_2^c\} \in E$ if they occur in one of the haplotypes either in forward or reverse order. More formally, there is an adjacency edge $\{m_1^b, m_2^c\} \in E$ if and only if there exists a sequence $A \in {\mathcal {H}}\cup \overline{{\mathcal {H}}}$ with $A =..m_1m_2..$ such that $m_1 = (m_1^a, m_1^b)$, $m_2 = (m_2^c, m_2^d)$ and $\{a,b\} = \{c, d\} = \{\text {t}, \text {h}\}$.

#### Example 2

**Fig. 2:**
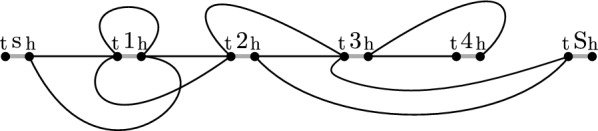
Illustration of variation graph from Example 2

Let $H_1 = {\texttt {s}\overline{\texttt {1}}{} \texttt {23}\overline{\texttt {4}}\overline{\texttt {3}}{} \texttt {S}}$, $H_2 = {\texttt {s111234}\overline{\texttt {3}}{} \texttt {S}}$, $H_3 = {\texttt {s}\overline{\texttt {1}}{} \texttt {23}\overline{\texttt {432}}{} \texttt {3}\overline{\texttt {43}}{} \texttt {S}}$, and $H_4 = {\texttt {s}\overline{\texttt {1}}{} \texttt {2S}}$, then the variation graph $G_{\mathcal {H}}$ of ${\mathcal {H}}= \{H_1, H_2, H_3, H_4\}$ is as illustrated in Fig. 2, with marker edges drawn in gray and adjacency edges in black.

#### Proposition 1

Let $G_{\mathcal {H}}$ be the variation graph of haplotypes ${\mathcal {H}}$, and ${\mathcal {X}}$ the set of all walks between terminal markers $s^\text {t}$ and $S^\text {h}$ in $G_{\mathcal {H}}$ with edges alternating between *E* and $\overrightarrow{E}$, then ${{\,\textrm{span}\,}}({\mathcal {H}}) = {\mathcal {X}}$.

#### Proof

$\subseteq$ Observe that no recombination can create a new pair of consecutive markers $m_1m_2$ that is not contained in any sequence $A \in {\mathcal {H}}\cup \overline{{\mathcal {H}}}$. Therefore, each haplotype $B \in {{\,\textrm{span}\,}}({\mathcal {H}})$ is a succession of consecutive markers drawn from sequences in ${\mathcal {H}}\cup \overline{{\mathcal {H}}}$, i.e., *B* can be delineated in $G_{\mathcal {H}}$ by following adjacency edges corresponding to its succession of consecutive markers.

$\supseteq$ Given an alternating walk $X = (s^\text {t}, s^\text {h}, \ldots , S^\text {t}, S^\text {h}) \in {\mathcal {X}}$, we show how to express *X* as a series of recombination events:

- Pick some haplotype $A \in {\mathcal {H}}$ and initialize $i \leftarrow 2$;
- Let $B \in {\mathcal {H}}\cup \overline{{\mathcal {H}}}$ be a sequence such that for some position *j*, $B[j..j+1] = m_1m_2$ with $m_1 = X[i-1..i]$ and $m_2 = X[i+1..i+2]$. Then $A \leftarrow \chi (A, B, i/2, j)$.
- Increase *i* by 2 and repeat step **b** unless $i=|X|-2$.

Observe that by construction of the variation graph $G_{\mathcal {H}}$, a suitable sequence $B \in {\mathcal {H}}\cup \overline{{\mathcal {H}}}$ must exist in each iteration of step **b**.$\square$

*Defining flows on variation graphs.* We determine a minimum set of founder sequences by solving a network flow problem in variation graph $G_{\mathcal {H}}$ where flow is allowed to travel along adjacency edges in either direction. Algorithm 1 describes the network flow problem. Each node is associated with two capacities corresponding to incoming and outgoing flow
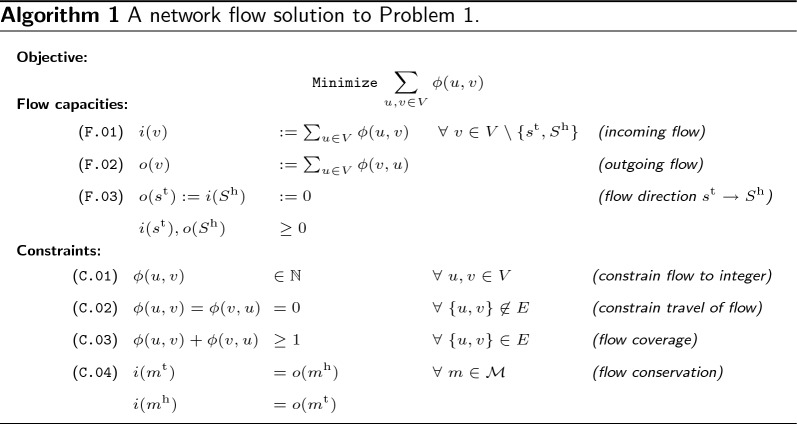
We then find a non-negative flow $\phi : V \times V \rightarrow {\mathbb {N}}$ such that the total flow $\sum _{u, v \in V} \phi (u, v)$ of graph $G_{\mathcal {H}}$ is minimized and satisfies constraints. Note that the flow can travel in both directions of an edge $\{u, v\} \in E$ and that $\phi (u, v) = \phi (v, u)$ does not hold true in general.

#### Example 2

**Fig. 3:**
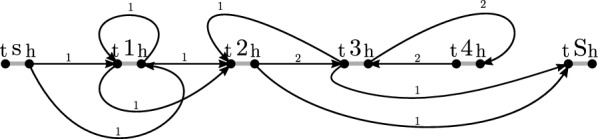
Network flow solution on variation graph $G_{\mathcal {H}}$ of Example 2

(cont’d) The drawing in Fig. 3 illustrates a flow solution on variation graph $G_{\mathcal {H}}$, with the direction and amount of flow along adjacency edges indicated by labeled arrowed arcs.

#### *Deriving haplotypes from flows.*

 By applying the Flow Decomposition Theorem [37, p. 80f], any *flow*, i.e., solution to the above-specified constraints, is decomposable into a set of alternating paths going from source $s^\text {t}$ to sink $S^\text {h}$ and a set of alternating cycles. Ahuja et al. [37] give a simple and efficient algorithm that does so in polynomial time and which we describe below, adapted to our circumstances. The idea is to perform a random walk in the graph from source to sink or within a cycle, thereby consuming flow along adjacency edges until all flow is depleted. The proof of the algorithm remains unchanged to that given by Ahuja et al., thus is not repeated here.

1. Set $$u \leftarrow s^\text {t}$$.
2. Each node is adjacent to exactly one other node through a marker edge. Setting out from current node *u*, traverse this incident marker edge to some node *v*, choose any neighbor *w* of *v* for which $\phi (v, w) > 1$. Follow the adjacency edge to *v* and decrease the flow $\phi (v, w)$ by 1. Set $u \leftarrow w$.
3. As long as $u \ne S^\text {t}$ do as follows: if *u* has been visited in the traversal before, then extract the corresponding alternating cycle from the recorded sequence and report it. Proceed with the traversal by repeating Step 2.
4. However, if $u = S^\text {t}$, follow the marker edge to $S^\text {h}$ and report the recorded sequence as a path.
5. If $s^\text {h}$ is incident with edges with positive flow, proceed with Step 1. Otherwise, there still might be strictly positive flow remaining in the graph corresponding to unreported cycles. In that case, pick any node $u \leftarrow m^a$ such that for some node *w*, $\phi (m^b, w) > 0$, $\{a, b\} = \{\text {t}, \text {h}\}$ and $m \in {\mathcal {M}}$, and proceed with Step 2 in order to report the next cycle.

#### Example 2

(cont’d) The components of the flow solution on variation graph $G_{\mathcal {H}}$ comprise two cycles C1 and C2, and two $(s^t, S^h)$-paths P1 and P2 are illustrated in Fig. 5.

What remains is the integration of cycles into walks that then correspond to the haplotypes of the founder set. The integration is facilitated by a graph structure, the *component graph*. The component graph $G' = (V', E', l)$ is an edge-labeled, directed multigraph, where, in its initial construction, each alternating $(s^\text {t}, S^\text {h})$-path and each cycle reported during flow decomposition is represented by a distinct node of $V'$. In the component graph $G'$, each cycle *c* of the flow decomposition sharing one or more markers with another component $c'$ is connected by one or more directed edges $(c, c')$ to that component, with each edge’s label $l(c, c')$ corresponding to one distinct shared marker, oriented according to the their succession in *c* (which may not be the same as in $c'$). The component graph is then successively deconstructed until empty as follows:

1. Remove and report all $(s^\text {t}, S^\text {h})$-walks with in-degree 0 from node set $V'$. Note that by construction, $(s^\text {t}, S^\text {h})$-walks have out-degree 0, i.e., those with in-degree 0 are singleton in $G'$.
2. Pick a cycle $c \in V'$ with in-degree 0, or, if none such exists, any arbitrary cycle $c \in V'$.
3. Pick an outgoing edge $(c, c') \in E'$ such that $c'$ is a $(s^\text {t}, S^\text {h})$-walk. If no such $c'$ exists, *c* is only adjacent to cycles, out of which one $c'$ is picked arbitrarily. Let $(m^a, m^b) \leftarrow l(c, c')$, $\{a, b\} = \{\text {t}, \text {h}\}$. If marker *m* is embedded in $c'$ in same orientation, i.e. $c' =..m^am^b..$, then linearize *c* in *m*, i.e., $c = m^b c_1.. c_{k-1} m^a$, and integrate it into $c'$ such that $c' \leftarrow .. m^a m^b c_1.. c_{k-1} m^a m^b..$ . Otherwise, integrate the reversed linearization of *c*, i.e, $c' \leftarrow .. m^b m^a c_{k-1}.. c_1 m^b m^a..$ . Remove cycle *c* and its outgoing edges from component graph $G'$.
4. Proceed with step 1 until no more components remain and all $(s^\text {t}, S^\text {h})$-walks are reported.

The search for components with in-degree 0 can be efficiently implemented through preorder traversal of $G'$. Note that each cycle must have at least one outgoing edge and that ultimately all cycles *must be* integrable into a $(s^\text {t}, S^\text {h})$-walk, otherwise this would imply that $G_{\mathcal {H}}$ contains a disconnected, circular component that is not reachable by an alternating path from source $s^\text {t}$ to sink $S^\text {h}$, thus contradicting the correctness of $G_{\mathcal {H}}$’s construction. The reported $(s^\text {t}, S^\text {h})$-walks represent the wanted haplotypes of a founder set.

#### Example 2

**Fig. 4:**
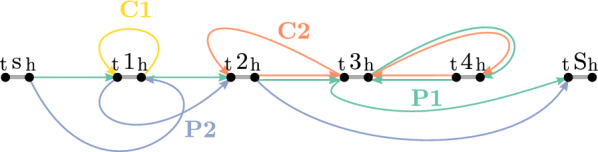
Component graph of components C1, C2, P1, and P2 (left) and a founder set of ${\mathcal {H}}$ (right) from Example 2

(cont’d) The plots in Fig. 4 depict the component graph of components C1, C2, P1, and P2 (left) and the final two $(s^t, S^h)$-walks that collectively represent a founder set of ${\mathcal {H}}$ (right).

**Fig. 5:**
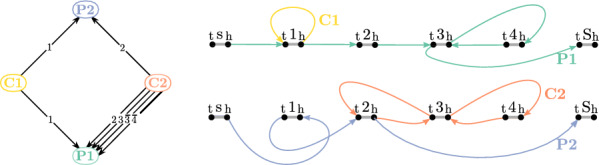
Components of flow solution on variation graph $G_{\mathcal {H}}$ of Example 2

We define the multiplicity of a consecutive marker pair $m_1m_2$, for any $m_1, m_2 \in {\mathcal {M}}\cup \overline{{\mathcal {M}}}$, as the number of times it appears as segment in forward or reverse order in a set of sequences ${\mathcal {X}}$ and introduce the following function for its retrieval:

$$\begin{aligned} {\mu _{{\mathcal {X}}}}(m_1, m_2) = |\{(A, i)\mid A[i..i+1]=m_1m_2 \text { or } A[i..i+1]=\overline{m_2m_1}: A \in {\mathcal {X}}\}| \end{aligned}$$

#### Theorem 1

Let ${\mathcal {F}}\subseteq {{\,\textrm{span}\,}}({\mathcal {H}})$, ${\mathcal {F}}$ is a solution to Problem 1 if and only if ${\mu _{\mathcal {F}}}$ corresponds to a minimum network flow in $G_{\mathcal {H}}$.

#### Proof

$\Rightarrow$ Any flow of variation graph $G_{\mathcal {H}}= (V, E)$ is decomposable into a set of haplotypes ${\mathcal {X}}$, as demonstrated above. Observe that the above-listed flow constraints enforce the derived haplotypes ${\mathcal {X}}$ to cover the entire graph $G_{\mathcal {H}}$ and consequently $G_{{\mathcal {X}}} = G_{\mathcal {H}}$. This implies that ${{\,\textrm{span}\,}}({\mathcal {X}}) = {{\,\textrm{span}\,}}({\mathcal {H}})$. Further, the total number of consecutive markers in a haplotype sequence *A* equals $|A|-1$ and therefore solutions to the specified network flow problem minimize quantity $\sum _{u, v \in V} \phi (u, v) = \sum _{A \in {\mathcal {X}}} |A| - |{\mathcal {X}}|$. This is equivalent to minimizing $\sum _{A \in {\mathcal {X}}} |A|$, because it is not possible to reduce the founder set size by concatenating two or more founder sequences without increasing the number of consecutive markers by an equal amount. Conversely, in the network flow specification, the sink node has no outgoing flow to the source node and therefore any founder set derived by a flow solution cannot be reduced by concatenation.

$\Leftarrow$We show that every founder set is also a solution to the specified minimum network flow problem. Assume that ${\mathcal {F}}$ is a founder set of haplotypes ${\mathcal {H}}$ and observe that multiplicities ${\mu _{\mathcal {F}}}$ correspond to a valid flow $\phi$ in $G_{\mathcal {H}}$. Now assume that there exists another flow $\phi '$ such that $\sum _{u, v \in V} \phi '(u, v) < \sum _{u, v \in V} \phi (u, v) = \sum _{A \in {\mathcal {F}}} |A| - |{\mathcal {F}}|$. Then, following the algorithm above, $\phi '$ can be decomposed into haplotype set ${\mathcal {F}}'$ such that $\sum _{A \in {\mathcal {F}}'} |A| - |{\mathcal {F}}'| < \sum _{A \in {\mathcal {F}}} |A| - |{\mathcal {F}}|$, contradicting the premise that ${\mathcal {F}}$ is a solution to Problem 1.$\square$

### Counting recombinations in founder sequences

We now provide a general algorithm for solving Problem 2. We show how this algorithm can be implemented to scale linearly with the input set of terminal sequences ${\mathcal {T}}$ and query sequence *Q* in time and space by utilizing generalized suffix trees. Supplementary Note N2 further describes a solution based on suffix arrays that has the same asymptotic runtime and space guarantees, but is considered more practical. Our approach builds on the concept that each terminal sequence *Q* that can be generated from set ${\mathcal {T}}$ is segmentable into a set of overlapping segments, where each such segment corresponds to a segment in a terminal sequence of ${\mathcal {T}}$. We call these segments ${\mathcal {T}}$-*blocks* for the remainder of this manuscript.

#### Lemma 2

*Q* can be generated from terminal sequences ${\mathcal {T}}$ if and only if *Q* is segmentable into a sequence of overlapping ${\mathcal {T}}$-blocks ${\mathcal {D}}=\{Q[..i_1], Q[i_1..i_2],..,Q[i_n..]\}$, with $1< i_1<..< i_n < |Q|$, i.e., for each $D \in {\mathcal {D}}$, $\exists ~A \in {\mathcal {T}}\cup \overline{{\mathcal {T}}}$ with $D \lhd A$.

#### Proof

$\Rightarrow$ If *Q* can be generated from ${\mathcal {T}}$ then there exists a series of recombinations $Q_1 \leftarrow \chi (A_1, A_2, i_1, k_1)$, $Q_2 \leftarrow \chi (Q_1, A_3, i_2, k_2)$,.., $Q_m \leftarrow \chi (Q_{m-2}, A_m, i_m, k_m)$ such that $A_1,..,A_m \in {\mathcal {T}}\cup \overline{{\mathcal {T}}}$ and $Q_m = Q$. Consequently, *Q* can be segmented into the set of overlapping ${\mathcal {T}}$-blocks ${\mathcal {D}} = \{A_1[..i_1], A_2[k_1..k_1+i_2-i_1],.., A_m[k_m..] \}$. $\Leftarrow$

For a given segmentation ${\mathcal {D}} = \{D_1,.., D_n\}$, *Q* is generated by a series of recombinations from ${\mathcal {T}}$ as follows: Let $Q_1[..i_1] = D_1$, $Q_1 \in {\mathcal {T}}\cup \overline{{\mathcal {T}}}$; For each *x* in 2..*n*, $Q_x \leftarrow \chi (Q_{x-1}, A_x, i_x, k)$ where $A_x[k..k+i_x-i_{x-1}] = D_x$, $A_x \in {\mathcal {T}}$; Observe that $Q_n = Q$.$\square$

Finding a minimum ${\mathcal {T}}$-block segmentation is equivalent to computing the minimum number of recombinations—the former differs in size from the latter only by an increment of 1. The recursive function $R_{\mathcal {T}}: \Theta \rightarrow {\mathbb {N}}$ defined below calculates the number of recombinations to generate query sequence *Q* from terminal sequences ${\mathcal {T}}$ by moving from one maximal ${\mathcal {T}}$-block of *Q* to the next. To this end, we define $L_{{\mathcal {T}}}(Q)$ as the length of the longest prefix of *Q* that is a ${\mathcal {T}}$-block, i.e., $L_{{\mathcal {T}}}(Q):= \arg \max _k \left\{ Q[..k] \lhd A \mid A \in {\mathcal {T}}\cup \overline{{\mathcal {T}}}\right\}$.

1 $$\begin{aligned} R_{\mathcal {T}}(Q) = {\left\{ \begin{array}{ll} 0 &{} \text {if } |Q| = L_{{\mathcal {T}}}(Q) \\ \infty &{} \text {else if }L_{{\mathcal {T}}}(Q) \le 1\\ 1 + R_{\mathcal {T}}(Q[L_{{\mathcal {T}}}(Q)..]) &{} \text {otherwise}\\ \end{array}\right. } \end{aligned}$$

$R_{\mathcal {T}}(Q) = \infty$ indicates that *Q* cannot be generated from ${\mathcal {T}}$. We prove that the algorithm is optimal, i.e., computes the minimum number of recombinations:

#### Proof

**Fig. 6:**
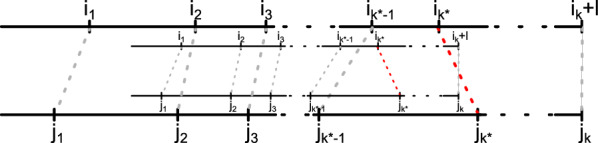
Illustration of the contradictory claim a shorter sequence of ${\mathcal {T}}$-blocks can be constructed than found by Eq. 1. The red dashed line indicates the contradictory situation that $i_{k^\star } < j_{k^\star }$. In that case $Q[i_{k^\star -1}..j_{k^\star }]$ would have been chosen as longest ${\mathcal {T}}$-block in recursion step $k^\star -1$

We prove this by induction over the number of recombinations identified by Eq. 1. Note that the total number of recombinations is bounded by the length of query *Q*. We show that for every *k* with $0 \le k < |Q|$ that $R_{\mathcal {T}}(Q[..i_k])$ reports the minimum number of recombinations for sequence $Q[..i_k]$.

(IB) In iteration $k=0$, $R(\cdot )$ receives the full-length query sequence *Q* and chooses the longest prefix of query *Q* that is a ${\mathcal {T}}$-block. It is clear that this is an optimal choice, since choosing a smaller prefix can only increase the number of recombinations. Note that if $l \le 1$, *Q* cannot be generated from ${\mathcal {T}}$ and *R*(*Q*) returns $\infty$.
(IS) Let $Q[i_{k-1}..i_k]$ be the ${\mathcal {T}}$-block identified in the *k*-th recurrence of $R_{\mathcal {T}}$ with the current query sequence being $Q[i_k..]$. In step $k+1$, $R_{\mathcal {T}}$ will again identify the segment $D = Q[i_k..i_k+l]$ of maximal length *l* that is a ${\mathcal {T}}$-block.
  Let us now claim that there is an shorter sequence of ${\mathcal {T}}$-blocks $Q[j_1..j_2],Q[j_2, j_3], \ldots Q[j_{k-1}, j_{k}]$ and $i_k+l = j_{k}$, as illustrated in Fig. 6. Then there must be some $0 \le k^\star \le k$ for which $j_{k^\star } > i_{k^\star }$. But if there were indeed a ${\mathcal {T}}$-block $Q[j_{k^\star -1}..j_{k^\star }]$, then $Q[i_{k^\star -1}..j_{k^\star }]$ is a suffix of $Q[j_{k^\star -1}..j_{k^\star }]$ and that would be the longest common prefix chosen by $R(\cdot )$ in iteration $i_{k^\star -1}$, contradicting the definition of $R(\cdot )$. Therefore, a shorter sequences of ${\mathcal {T}}$-blocks cannot exist.

$$\square$$

The algorithm can efficiently count recombinations by utilizing the *suffix tree* data structure [38]. To this end, the suffix tree is constructed on sequence $$T\mathtt {\$}$$ corresponding to a concatenation of terminal sequences $${\mathcal {T}}\cup \overline{{\mathcal {T}}}$$ of any given order, terminated by sentinel “$$\mathtt {\$}$$”. In doing so, we assume that terminal markers $$s, \overline{s}, S$$, and $$\overline{S}$$ abide lexicographic order $ $$< \{S, {\overline{s}}\}< \{s, {\overline{S}}\} < m~\forall ~m \in {\mathcal {M}}\cup {\overline{{\mathcal {M}}}}$$. Suffix trees can be constructed in linear time and space [39], and matching substrings in $$T\mathtt {\$}$$ can be performed in time linear to the length of the matching. To assess the time complexity of the recursion, observe that $$R_{\mathcal {T}}(Q)$$ is recursed at most $$|Q|-1$$ times, if all $${\mathcal {T}}$$-blocks have length 2. We conclude:

#### Theorem 3

Problem 2 is solvable in $O(|{\mathcal {T}}| + |Q|)$ time and space.

### Minimizing recombinations in founder sequences

We now present an algorithm towards solving Problem 3, i.e., the problem of finding a founder set that minimizes the number of recombinations needed for its construction from a given set of haplotypes ${\mathcal {H}}$. Solving this problem requires the simultaneous computation of solutions to both the Founder Set and the Recombination Count problem and constitutes in combing through an exponentially large search space. We simplify the problem by presuming that the multiplicities of consecutive marker pairs in a solution to the Parsimonious Founder Set Problem are also optimal under the Founder Set problem. In other words, our approach is exact under the assumption that the overall multiplicity of each pair of consecutive markers in a founder set that is a solution to Problem 3 is known, yet the pair’s particular orientation and location in the founder sequences are not. To this end, we presume a function ${{\hat{\mu }_{\mathcal {F}}}}(m_1, m_2)$ acting as oracle for the overall multiplicity of any given pair of consecutive oriented markers $m_1, m_2 \in {\mathcal {M}}\cup \overline{{\mathcal {M}}}$ in a solution ${\mathcal {F}}$ to Problem 3. More specifically, ${{\hat{\mu }_{\mathcal {F}}}}(m_1, m_2)$ reports the total number of occurrences of $m_1m_2$ and $\overline{m_2m_1}$ in founder set ${\mathcal {F}}$. Note that our experiments directly use the results of Problem 1 as input for Problem 3, i.e., ${{\hat{\mu }_{\mathcal {F}}}}(m_1, m_2)$ reports the number of occurrences of $(m_1, m_2)$ in a solution to Problem 1. This makes our experimental solutions to Problem 3 heuristic. In addition, we make use of function ${\hat{\gamma }_{\mathcal {F}}}(m):= \sum _{m' \in {\mathcal {M}}\cup \overline{{\mathcal {M}}}} {{\hat{\mu }_{\mathcal {F}}}}(m, m')$ to retrieve the multiplicity of any marker $m \in {\mathcal {M}}\cup \overline{{\mathcal {M}}}$. Note that ${{\hat{\mu }_{\mathcal {F}}}}$ and ${\hat{\gamma }_{\mathcal {F}}}$ are symmetric with respect to the relative orientation of markers, ${{\hat{\mu }_{\mathcal {F}}}}(m_1, m_2) = {{\hat{\mu }_{\mathcal {F}}}}(\overline{m_2}, \overline{m_1})$ and ${\hat{\gamma }_{\mathcal {F}}}(m) = {\hat{\gamma }_{\mathcal {F}}}(\overline{m})$. Our solution makes use of the *flow graph* that is defined in the subsequent paragraph. We calculate a matching in the flow graph that describes a set of founder sequences, each corresponding to a succession of segments of haplotypes ${\mathcal {H}}$. The objective of the matching is to minimize the total number of ${\mathcal {H}}$-blocks across all founder sequences which is equivalent to minimizing the number of recombinations for their construction from haplotype set ${\mathcal {H}}$.

*Flow graph construction.* The flow graph $G_{{\mathcal {H}}, {{\hat{\mu }_{\mathcal {F}}}}} = (V_{{\hat{\mu }_{\mathcal {F}}}}, E_{{\hat{\mu }_{\mathcal {F}}}}\cup \overrightarrow{E_{{\hat{\mu }_{\mathcal {F}}}}})$ is a directed edge-colored multigraph with adjacency edges $E_{{\hat{\mu }_{\mathcal {F}}}}$ and marker edges $\overrightarrow{E_{{\hat{\mu }_{\mathcal {F}}}}}$, where each marker extremity $m^a$ with $m \in {\mathcal {M}}$ and $a \in \{\text {t}, \text {h}\}$, gives rise to $2\cdot {\hat{\gamma }_{\mathcal {F}}}(m)$ elements in node set $V_{{\hat{\mu }_{\mathcal {F}}}}$, representing ${\hat{\gamma }_{\mathcal {F}}}(m)$ many *in* (i) and ${\hat{\gamma }_{\mathcal {F}}}(m)$ many *out* (o) nodes. Hence, each node in the flow graph is represented by a triple of the form $\{\text {i}, \text {o}\} \times {\mathcal {M}}^{\text {t}} \cup {\mathcal {M}}^{\text {h}} \times \mathbb N$ with the complete vertex set being $V_{{\hat{\mu }_{\mathcal {F}}}}= \{(\text {i}, m^a, x) \mid m \in {\mathcal {M}}, a \in \{\text {t}, \text {h}\}, x \in 1..{\hat{\gamma }_{\mathcal {F}}}(m) \} \cup \{(\text {o}, m^a, x) \mid m \in {\mathcal {M}}, a \in \{\text {t}, \text {h}\}, x \in 1..{\hat{\gamma }_{\mathcal {F}}}(m) \}$. Each out node $u \in V_{{\hat{\mu }_{\mathcal {F}}}}{\setminus } (\{(\text {i}, S^\text {h}, x) \mid 1..{\hat{\gamma }_{\mathcal {F}}}(S)\} \cup \{(\text {o}, s^\text {t}, x) \mid 1..{\hat{\gamma }_{\mathcal {F}}}(s)\})$ is incident with *one and only one* directed adjacency edge (*u*, *v*) connecting *u* to some in node *v* thereby realizing one occurrence of its representing pair of consecutive oriented markers in a founder sequence. Conversely, each forward-oriented marker $m \in {\mathcal {M}}$ contributes ${\hat{\gamma }_{\mathcal {F}}}(m)^2$ many directed marker edges that connect in/tail nodes with out/head nodes, i.e., $\{((\text {i}, m^\text {t}, x), (\text {o}, m^\text {h}, y)) \mid x, y \in 1..{\hat{\gamma }_{\mathcal {F}}}(m)\}$. Analogously, each reverse-oriented marker $\overline{m} \in \overline{{\mathcal {M}}}$ contributes ${\hat{\gamma }_{\mathcal {F}}}(m)^2$ many in/head-to-out/tail-directed marker edges $\{((\text {i}, m^\text {h}, x), (\text {o}, m^\text {t}, y)) \mid x, y \in 1..{\hat{\gamma }_{\mathcal {F}}}(m)\}$.

#### Example 2

**Fig. 7:**
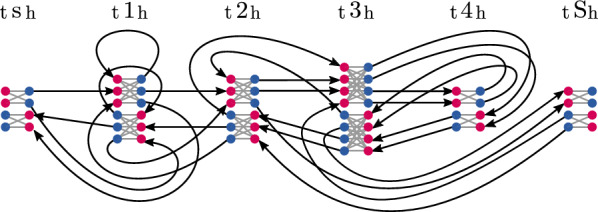
Flow graph $G_{{\mathcal {H}},{{\hat{\mu }_{\mathcal {F}}}}}$ of Example 2. In nodes and out nodes are highlighted in red and blue, respectively. For clarity, the direction of marker edges (gray edges; directed from in to out node) is omitted in the illustration

(cont’d) Fig. 7 visualizes the flow graph $G_{{\mathcal {H}},{{\hat{\mu }_{\mathcal {F}}}}}$ for the given set of haplotypes ${\mathcal {H}}= \{{\texttt {s}\overline{\texttt {1}}{} \texttt {23}\overline{\texttt {43}}{} \texttt {S}}$, ${\texttt {s111234}\overline{\texttt {3}}{} \texttt {S}}$, ${\texttt {s}\overline{\texttt {1}}{} \texttt {23}\overline{\texttt {432}}{} \texttt {3}\overline{\texttt {43}}{} \texttt {S}}$, ${\texttt {s}\overline{\texttt {1}}{} \texttt {2S}}\}$ and a given ${{\hat{\mu }_{\mathcal {F}}}}$.

*Graph decomposition.* A perfect matching of marker edges in flow graph $G_{{\mathcal {H}}, {{\hat{\mu }_{\mathcal {F}}}}}$ produces a set of alternating walks and alternating cycles through $G_{{\mathcal {H}},{{\hat{\mu }_{\mathcal {F}}}}}$, yet only half of the graph is eligible to form a solution to Problem 3. More precisely, for each marker $m \in {\mathcal {M}}$, exactly half of the number of its associated nodes in $V_{{\hat{\mu }_{\mathcal {F}}}}$ must be *saturated*, i.e., incident with a matching edge. The other half as well as their incident edges must remain unsaturated. Further, we aim to admit only matchings that consist entirely of alternating $((\text {i}, s^\text {t}, x), (\text {o}, S^\text {h}, y))$-walks, because only those correspond to valid haplotypes of ${{\,\textrm{span}\,}}({\mathcal {H}})$.

At last, we aim to assign to each saturated node $v \in V_{{\hat{\mu }_{\mathcal {F}}}}$ a position in some haplotype *A* of given haplotype set ${\mathcal {H}}$. That way, we are able to determine whether the incident adjacency edge serves as continuation of the associated haploblock of *A*, or whether the incident saturated marker edge implies a recombination between two distinct ${\mathcal {H}}$-blocks.

The *Integer Linear Program* shown in Algorithm 2 implements the above-stated constraints.

#### Example 2

**Fig. 8:**
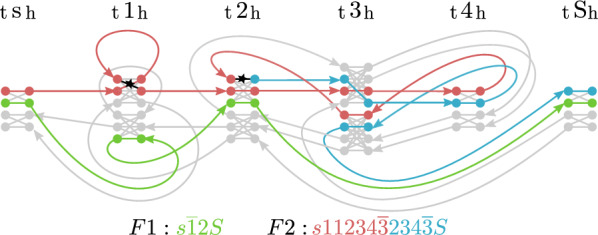
Solution to Algorithm 2 for $G_{{\mathcal {H}},{{\hat{\mu }_{\mathcal {F}}}}}$ for Example 2

(cont’d) Fig. 8 illustrates a matching that is solution to Algorithm 2 for $G_{{\mathcal {H}},{{\hat{\mu }_{\mathcal {F}}}}}$. The founder sequences are spelled out on the bottom, colored by haplotype (red, blue and green for haplotypes 2, 3 and 4 respectively). Unsaturated nodes and edges are grayed out, haplotype assignments implied by colored paths. The solution features two recombinations, marked by “$\star$” along their associated marker edges.

*Objective.* The ILP maximizes the sum over all $\texttt {t}$ variables, which corresponds to finding a set of founder sequences that has a maximum number of marker pairs $m_1m_2$ associated with consecutive positions in any of the haplotypes ${\mathcal {H}}$. Conversely, any marker pair that is not linked to a position in a haplotype of ${\mathcal {H}}$ represents a recombination event.

*Matching constraints.* Each edge $(u, v) \in E_{{\hat{\mu }_{\mathcal {F}}}}\cup \overrightarrow{E_{{\hat{\mu }_{\mathcal {F}}}}}$ and node $w \in V_{{\hat{\mu }_{\mathcal {F}}}}$ of flow graph $G_{{\mathcal {H}}, {{\hat{\mu }_{\mathcal {F}}}}}$ is associated with binary variables of $\texttt {x}(u, v)$ and $\texttt {y}(w)$, respectively, that determine their saturation in a solution (cf. domains D.1 and D.2). Constraint C.01 ensures that each saturated marker edge is incident with saturated nodes. Perfect matching constraints, i.e., constraints that impose each saturated node being incident with exactly one marker edge, are implemented by constraint C.02. Similarly, constraint C.03 ensures that an adjacency edge is saturated if and only if its incident nodes are saturated. In other words, constraints C.01-C.03 together ensure that each component of the saturated graph corresponds to an alternating path or cycle component (the latter being prohibited by further constraints). The following two constraints C.04 and C.05 control the overall size of the saturated graph. In doing so, they ensure that, in a solution to Problem 3, the number of saturated nodes and adjacency edges matches the postulated multiplicity of markers ${\hat{\gamma }_{\mathcal {F}}}(m)$, $m\in {\mathcal {M}}\cup \overline{{\mathcal {M}}}$, and pairs of consecutive markers ${{\hat{\mu }_{\mathcal {F}}}}(m_1, m_2)$, $m_1, m_2 \in {\mathcal {M}}\cup \overline{{\mathcal {M}}}$, respectively.

*Path constraints.* Constraints C.05-C.08 force each component of the saturated graph to start and end in nodes associated with source $s^\text {t}$ and sink $S^\text {h}$, respectively, thereby ruling out any cycles. To this end, they make use of a set of integer variables $\texttt {f}(v)$ over all vertices $v \in V_{{\hat{\mu }_{\mathcal {F}}}}$ (cf. Domain D.03) that define an increasing flow within each saturated component that is bounded by constant *T* corresponding to the total flow of the graph, i.e., $T:= \sum _{m \in {\mathcal {M}}} {\hat{\gamma }_{\mathcal {F}}}(m)$. In each saturated marker edge, the flow is increased by 1 while along each adjacency edge, flow is kept constant. This prevents the formation of saturated cycles, because their flow would be infinite. Lastly, constraint C.08 preclude paths from starting in $S^\text {h}$ or ending in $s^\text {t}$, leaving only one option for any saturated component open, that is, the formation of a $(s^\text {t}, S^\text {h})$-path.

*Haplotype assignment.* Each node $v \in V_{{\hat{\mu }_{\mathcal {F}}}}$ in a solution to the ILP is associated with exactly one position $j \in 1..|A|$ in a haplotype *A* of ${\mathcal {H}}$, recorded by binary variables $\texttt {c}(A[j], v)$.Q Moreover, any marker edge whose incident pair of nodes is associated with the same position of the same haplotype corresponds to a ${\mathcal {H}}$-block, i.e, no recombination within this marker has taken place. Each marker edge $(u, v) \in \overrightarrow{E_{{\hat{\mu }_{\mathcal {F}}}}}$ that is linked by the ILP solver to a position *j* in a haplotype $A \in {\mathcal {H}}$ contributes a score unit to the objective function. These score units are encoded by binary variables $\texttt {t}(A[j], u, v)$ (cf. domain D.05). Constraint C.09 ensures that each marker is associated with exactly one position *j* in a haplotype *A* of set ${\mathcal {H}}\cup \overline{{\mathcal {H}}}$, while C.10 confines incident nodes of adjacency edges to represent a consecutive marker pair $A[j..j+1]$. At last, constraint C.11 allows $\texttt {t}$ variables of marker edges to take on value 1 only if that marker edge is saturated and its incident nodes are associated with the same haplotype position.
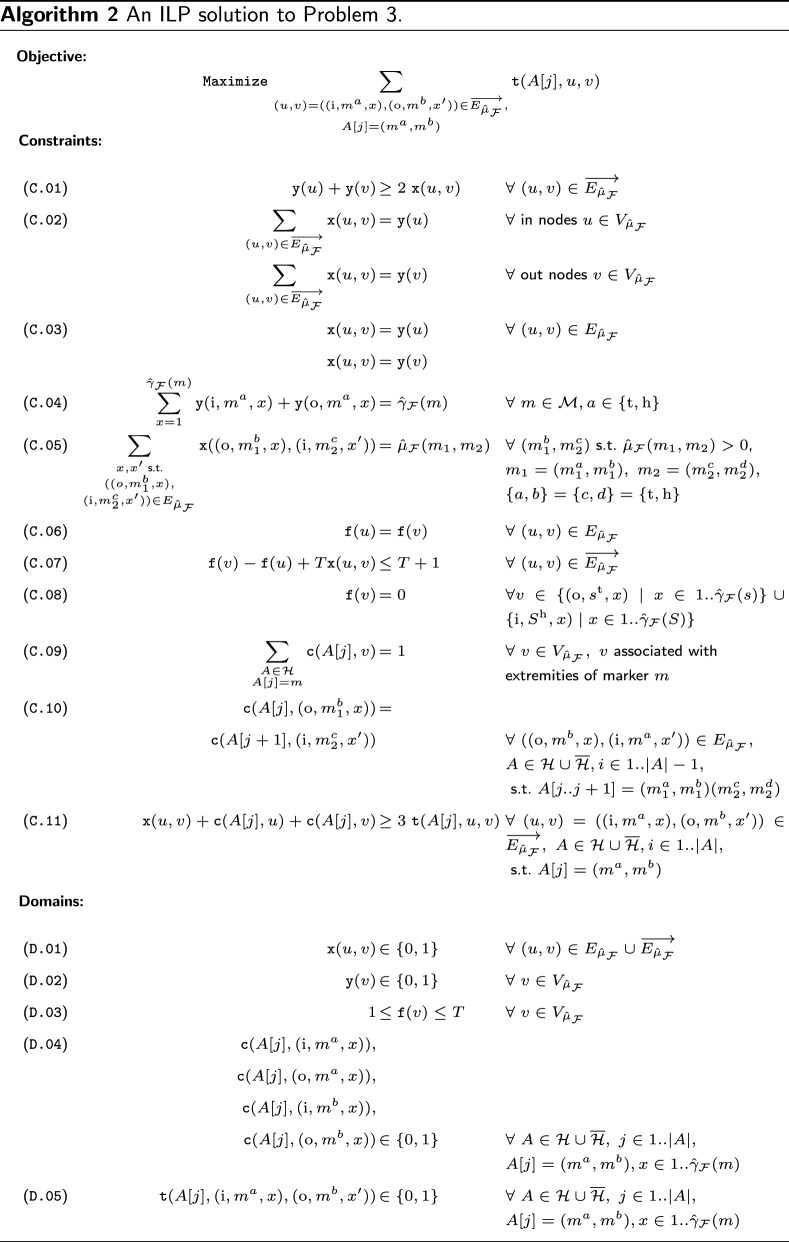


## Results

We implemented our methods in the programming language Rust [40] and used Gurobi [41] as the solver. Our software is *open source* and publicly available online [42]. To run Algorithm 2 on a given set of haplotypes ${\mathcal {H}}$, we estimated the overall multiplicity ${{\hat{\mu }_{\mathcal {F}}}}(m_1, m_2)$ of pairs of consecutive markers $m_1m_2$ from a network flow solution to Problem 1 on ${\mathcal {H}}$. Note that, because there is no guarantee that an optimal solution to Problem 3 exists that has also optimal flow under Problem 1, our approach does not guarantee exact solutions.

For benchmarking purposes, we ran Gurobi single-threaded and recorded wall clock time (in seconds) and *Proportional Set Size (PSS)* (in Megabytes (MB)) for memory usage. The choice of using PSS rather than measures such as *Resident Set Size (RSS)* or *Unit Set Size (USS)* is largely arbitrary, however all three measures were highly similar in all experiments and within 100 MB of each other at the extreme. Optimization time was capped at 30 min, beyond which the solver stops and returns its best-effort solution found thus far.

### Experimental data

We benchmarked the performance of our algorithms by conducting experiments on both simulated data and a real-world data set. The former presumed a simulator, capable of generating haplotypes with duplicated and inverted markers that can produce intricate homologous recombinations while providing control over the degree of complexity. To this end, we implemented our own simulation tool that constructs a single haplotype sequence sampled at random to serve as seed. This seed sequence is adjustable by the following parameters: (i) number of distinct markers, i.e., the size of its variation graph, (ii) ratio of duplications, i.e., the number of additional edges inducing duplications in a walk of the graph, (iii) ratio of inversions, i.e., the proportion of inverted orientations within the set of duplications, and lastly (iv) the number of haplotypes that are input to subsequent founder set reconstruction. The latter are generated by performing random walks in the seed sequence’s variation graph and retaining only those leading from source to sink. In doing so, our simulator does not report nor have knowledge of a true founder set. Our simulator, discussed in more detail in Supplementary Note N1, enables us to explore various parameterizations that match different situations in biological data.

One important point concerns co-optimality. Problems 1 and 3 do not guarantee a unique solution. In fact, the pool of co-optimal solutions is often large for both problems. One contributing factor to co-optimality are cycles that are shared across multiple haplotypes, because they can be integrated in different orders. Further, the solution does not provide any information that could enable one to generate all co-optimal solutions nor discern between them, making a measure of accuracy challenging, since there is no guarantee that the “correct” founder sequence(s) will be seen in any number of trials.

In addition to simulated data, we applied our methods on a biological data set from the human 1p36.13 locus described by Porubsky et al. [22] to demonstrate the computational performance on realistic instances.

### Simulation experiments

To assess the impact of parameter configurations on the results, we ran a number of different experiments wherein all but one parameters are fixed. A reasonable choice of constants seemed to be 100 distinct markers, $10\%$ of duplications, $10\%$ of inversions and 10 haplotypes, motivated by our data on the 1p36.13 locus (8 markers, 68 haplotypes, 57% of duplications) and statistics compiled by Porubsky et al. [22] ($6-7\%$ duplications in the whole genome, $<1\%$ inversions).

**Fig. 9:**
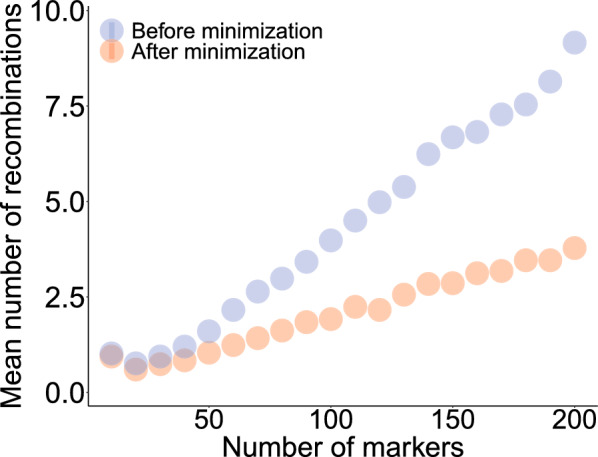
*Mean number of recombinations by the size of the graph.* Experiments were ran with values ranging from 10 to 200 in for the number of markers, in increments of 10. The ratio of duplications and of inversions was fixed to 10%, and number of haplotypes to 10. Each colored dot represents the mean number of recombinations over 50 replicates for one parameter set, after random assignment trials (blue) and after optimization (red)

*Reduction in number of recombinations.* To evaluate the efficacy of our solution to Problem 3, we compared the number of recombinations returned by Algorithm 2 to that in a solution obtained by our network flow algorithm for Problem 1. To this end, we set the output of Algorithm 1 against an implementation of a solution to Problem 2, described in further detail in Supplementary Note N2. Figure 9 summarizes the outcome of this experiment. Overall, Algorithm 2 found a solution with fewer recombinations in all instances but a few where Gurobi returned barely best-effort solutions after reaching the time limit of 30 min, all of which exhibited a gap of at least 100%. The parameter settings in those cases were extremal.

Across all experiments, the mean estimated number of recombinations increased linearly by approximately 1.7 per 100 markers after minimization, compared to 4.5 per 100 without it. The values reached respectively 3.8 and 9.1 at 200 markers. The simulations here were carried out with a fixed number of haplotypes and ratios of duplications and of inversions. Results for experiments with other variable parameters are shown in Additional file 1: Figure S1.

**Fig. 10:**
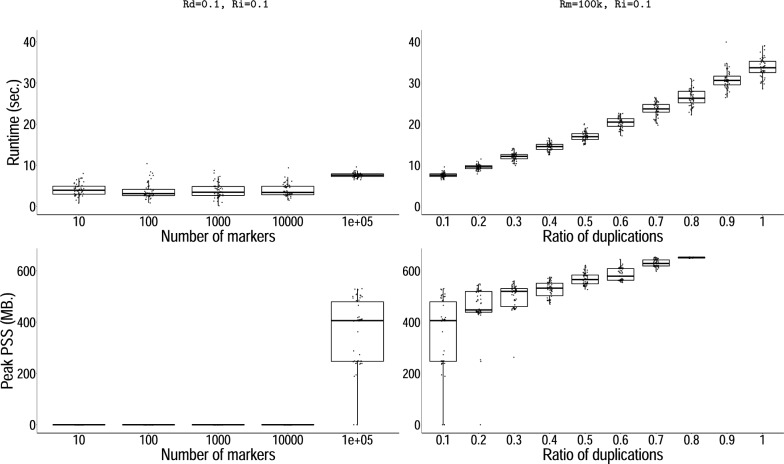
*Problem* 1, *flow computational performance benchmarks.* Runtime in seconds (upper panels) and peak PSS in MB (lower panels), as a function of the number of markers (left) and of the ratio of duplications (right). For each experiment, the remaining parameters are fixed as indicated above. The abbreviations read as follows: *Nm* number of markers; *Rd* ratio of duplications; and *Ri*, ratio of inverted duplications

*Flow solution benchmark.* Computing solutions with our network flow algorithm proved to be in almost all of our experiments near-instantaneous. By varying the number of distinct markers, the algorithm’s performance begins to deteriorate only with very large instances beyond 100k distinct markers and becomes excruciating for instances above 1M markers. When varying other parameters, we fixed the number of distinct markers to 100k rather than 100. Under 100k markers, execution completed after a mean wall clock time of $3.4\pm 2.0$ seconds. In $95\%$ of all experiments, the solver’s runtime was too short to make sufficient measurements for benchmarking memory usage; the maximum PSS for the remaining ones measured at 78 MB. Over the 100k mark, both the graph size and duplication ratio began to reduce performance, with an average runtime of $19.7\pm 8.7$s. The ratio of inversions on the other hand did not affect performance (Suppl. Figure S3). We measured peak memory consumption at 758 MB across all conditions, which also occurred only at the very extremes of 100k distinct markers and a 100% ratio of duplications (Fig. 10).

**Fig. 11:**
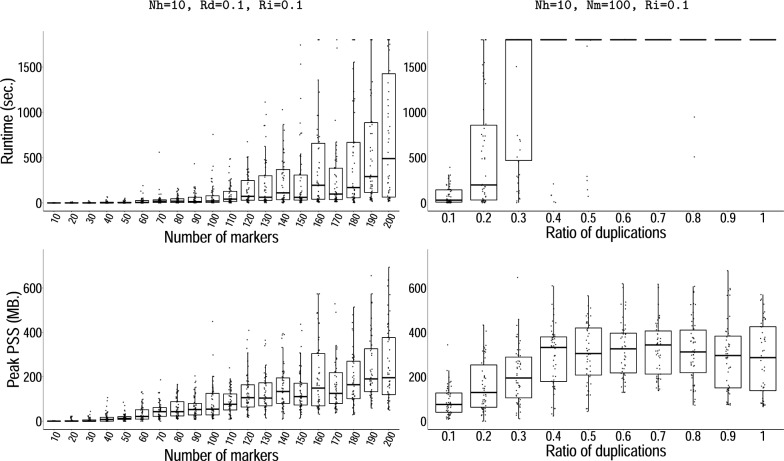
*Problem* 3, *recombinations minimization performance benchmarks.* Plots analogous to Fig. 10. Runtime in seconds (upper panels) and peak PSS in MB (lower panels), as a function of the number of markers (left) and of the ratio of duplications (right). For each experiment, the remaining parameters are fixed as indicated above. The abbreviations read as follows: *Nh* number of haplotypes; *Nm*, number of markers; *Rd* ratio of duplications; and *Ri*, ratio of inverted duplications

*Recombination minimization benchmark.* As shown previously, Algorithm 2 successfully reduces the number of recombinations in solutions to Problem 1. However, its runtime increased dramatically with only moderate increments of any but one parameter of our simulator, the ratio of inversions, which did not play any role in performance (Additional file 1: Figure S2). For the remaining three, going beyond instances of 200 distinct markers, 20% of duplications, or 40 haplotypes typically did not allow for the optimization to finish in a reasonable amount of time (Fig. 11, Additional file 1: Figure S2). A similar but much less pronounced trend was seen with memory usage, which still remained relatively low. Peak memory usage was again observed at extreme parameter values with a PSS of 1072 MB with 50 haplotypes.

### Application: locus 1p36.13

**Fig. 12:**
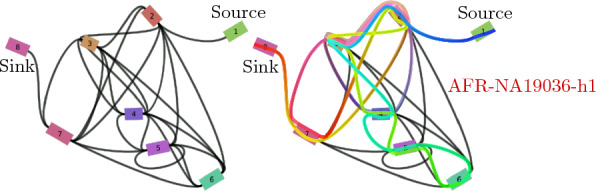
*Graphical representation of the variation graph for the 1p36.13 locus data.* On the left, a 2D plot rendered by Bandage [43]. Markers are represented as numbered colored rectangles, and the undirected edges connecting them as black curves. Markers 1 and 8 correspond respectively to the source and the sink of the graph. The right plot shows the walk through the graph from source (blue) to sink (red) corresponding to the sequence of haplotype AFR-NA19036-h1, a sample of African origin from our experimental data. The sample’s sequence in the previously established notation is: ${ \texttt {123}\overline{\texttt {4}}{} \texttt {56}\overline{\texttt {5432}}{} \texttt {7}\overline{\texttt {32432}}{} \texttt {78}}$

We obtained data from 68 human haplotypes (two per 34 individuals) at the 1p36.13 locus from Porubsky et al. [22] and the T2T-CHM13 human reference sequence [16]. The sequences comprise only eight distinct markers, terminal markers included. The sequences are attributed to five super populations, out of which 18 are of African origin (AFR), 16 of Eastern Asian (EAS), 12 of Admixed American (AMR), 12 of European (EUR), and 10 are South Asian (SAS). Their variation graph is densely connected with 26 edges (Fig. 12). The 68 haplotypes display a high degree of genetic diversity, with haplotype sequences differing in order, orientation, and copy number of the marker (Suppl. Table T1). Haplotype lengths in terms of the number of markers vary from 15 to 26, with a median of 19.

Our network flow algorithm determined that the data set can be generated from a single founder sequence. Our randomized algorithm for calculation of the minimum number of recombinations in a solution to Problem 1 asserted 15 recombinations after 1M trials, while Algorithm 1 obtained an optimal solution that revealed only 9 recombinations. Minimization completed in 60.3 s with a peak PSS of 225 MB. Note that there exists multiple other co-optimal solutions; Suppl. Figure S4 is an illustration of one.

## Discussion

The advent of sequencing technology and genome assembly methodology to reconstruct full human genomes enables research into previously inaccessible segmental duplication loci. This exciting opportunity entails a demand for explanatory models that can infer evolutionary relationships and histories of complex repetitive genomic regions. In this work, we propose a model capable of explaining a broad range of balanced and unbalanced genome rearrangements. Our experiments on simulated data and on the 1p36.13 locus demonstrate that our algorithmic solutions to the founder set problem and the problem of minimizing recombinations in founder sets are capable of processing realistic instances. While the complexity of Problem 3 remains undetermined, we conjecture it to be NP hard.

Importantly, the model we are proposing is based on a molecular mechanism with a well-established role in shaping segmental duplication architecture. In our view, many past models of genome rearrangements have not sufficiently captured biological reality and there is an important need for further research aiming to incorporate knowledge of molecular mechanisms into such models. For instance, we envision future models that additionally include mechanisms like non-homologous end joining (NHEJ) and mobile element insertions. Furthermore, actual rates at which NAHR occurs depend on factors like the length of the duplicated sequence, the sequence similarity, as well as the presence of specific sequence motifs. In our current approach, these aspects are only partially and indirectly captured through the graph construction process. We aim to address and model these factors explicitly in future work.

### Supplementary Information

**Additional file 1: Figure S1. **Reduction in the number of recombinations following minimization. The plots show the total number of recombinations before (blue dots) and after (red dots) minimization, as a function of each simulation parameter. **Figure S2. **Number of recombinations minimization benchmarks. Runtime (upper panels) and peak PSS (lower panels) as a function of the number of haplotypes (left) and the ratio of inverted duplications (right). **Figure S3. ** Flow computation performance with a variable ratio of inversions. Runtime (left) and memory usage (right) as a function of this parameter. **Figure S4. ** Visualization of a solution to the minimization problem on the 1p36.13 locus. The gray bars correspond to the graph’s nodes, labeled 1 to 8. The founder sequence (>1>2>3<7>5>2>3<4>5>5<6<4<3>7<3<2 <4>5>6<5>4<5<4<3 <2>7<3>6>7<3<4<3<2>6<4>3>2>7>8) is traced from top to bottom. A slanted line indicates the underlying node being traversed; if slanted rightwards, traversal is in forward direction, and if slanted leftwards, traversal is in reverse direction. Colors correspond to different haplotypes. The haplotype sequence is: EUR-HG00171-h2, AFR-NA19036-h1, SAS-GM20847-h2, AFR-HG03065-h2, AFR-NA19036-h1, AFR-NA19036-h1, AMR-HG01573-h2, AFR-HG02011-h2, AFR-HG03371-h2, SAS-HG03683-h2. Recombinations are marked with a star. **Figure S5. ** Reduction in the number of recombinations following minimization. The plots show the total number of recombinations before (blue dots) and after (red dots) minimization, as a function of each simulation parameter. **Table S1. ** Sorted haplotype marker sequences used for analyzing the 1p36.13 locus.

## Data Availability

All data used for the analysis of the 1p36.13 locus is included in Additional file 1: Table T1. The simulation experiments are solely based on data generated by a program named *hapsim*, available in source code form in the public repository [42].

## Abbreviations

DCJ: Double cut and join
HPRC: Human pangenome reference consortium
ILP: Integer linear program
LCA: Least common ancestor
MB: Megabytes
NAHR: Non-allelic homologous recombination
NHEJ: Non-homologous end joining
PSS: Proportional set size
RMQ: Range minimum query
RSS: Resident set size
SD: Segmental duplication
SNP: Single nucleotide polymorphism
SV: Structural variant
USS: Unit set size
