# Correction: Constructing founder sets under allelic and non-allelic homologous recombination

**DOI:** 10.1186/s13015-023-00244-0

**Published:** 2023-12-06

**Authors:** Konstantinn Bonnet, Tobias Marschall, Daniel Doerr

**Affiliations:** Institute for Medical Biometry and Bioinformatics, Medical Faculty, and Center for Digital Medicine, Heinrich Heine University, Moorenstr. 5, 40225 Düsseldorf, Germany

**Correction: Algorithms for Molecular Biology (2023) 18:15** **https://doi.org/10.1186/s13015-023-00241-3**

The Additional file 1 which originally published contained errors. It has now been replaced with the correct file.

The original article [1] has been corrected.

### Supplementary Information

**Additional file 1: Figure S1**. Reduction in the number of recombinations following minimization. The plots show the total number of recombinations before (blue dots) and after (red dots) minimization, as a function of each simulation parameter. **Figure S2**. Number of recombinations minimization benchmarks. Runtime (upper panels) and peak PSS (lower panels) as a function of the number of haplotypes (left) and the ratio of inverted duplications (right). **Figure S3**. Flow computation performance with a variable ratio of inversions. Runtime (left) and memory usage (right) as a function of this parameter. **Figure S4**. Visualization of a solution to the minimization problem on the 1p36.13 locus. The gray bars correspond to the graph’s nodes, labeled 1 to 8. The founder sequence (>1>2>3<7>5>2>3<4>5>5<6<4<3>7<3<2<4>5>6<5>4<5<4<3<2>7<3>6>7<3<4<3<2>6<4>3>2>7>8) is traced from top to bottom. A slanted line indicates the underlying node being traversed; if slanted rightwards, traversal is in forward direction, and if slanted leftwards, traversal is in reverse direction. Colors correspond to different haplotypes. The haplotype sequence is: EUR-HG00171-h2, AFR-NA19036-h1, SAS-GM20847-h2, AFR-HG03065-h2, AFR-NA19036-h1, AFR-NA19036-h1, AMR-HG01573-h2, AFR-HG02011-h2, AFR-HG03371-h2, SAS-HG03683-h2. Recombinations are marked with a star. **Table S1**. Sorted haplotype marker sequences used for analyzing the 1p36.13 locus.
