# Supplementary material for: Correction: Constructing founder sets under allelic and non-allelic homologous recombination
Source: Algorithms Mol Biol. 2023 Dec 6;18:20. doi: 10.1186/s13015-023-00244-0 (PMC10698948; doi:10.1186/s13015-023-00244-0)
Supplement: Supplementary file 1 — Additional file 1: Figure S1. Reduction in the number of recombinations following minimization. The plots show the total number of recombinations before (blue dots) and after (red dots) minimization, as a function of each simulation parameter. Figure S2. Number of recombinations minimization benchmarks. Runtime (upper panels) and peak PSS (lower panels) as a function of the number of haplotypes (left) and the ratio of inverted duplications (right). Figure S3. Flow computation performance with a variable ratio of inversions. Runtime (left) and memory usage (right) as a function of this parameter. Figure S4. Visualization of a solution to the minimization problem on the 1p36.13 locus. The gray bars correspond to the graph’s nodes, labeled 1 to 8. The founder sequence (>1>2>3<7>5>2>3<4>5>5<6<4<3>7<3<2<4>5>6<5>4<5<4<3<2>7<3>6>7<3<4<3<2>6<4>3>2>7>8) is traced from top to bottom. A slanted line indicates the underlying node being traversed; if slanted rightwards, traversal is in forward direction, and if slanted leftwards, traversal is in reverse direction. Colors correspond to different haplotypes. The haplotype sequence is: EUR-HG00171-h2, AFR-NA19036-h1, SAS-GM20847-h2, AFR-HG03065-h2, AFR-NA19036-h1, AFR-NA19036-h1, AMR-HG01573-h2, AFR-HG02011-h2, AFR-HG03371-h2, SAS-HG03683-h2. Recombinations are marked with a star. Table S1. Sorted haplotype marker sequences used for analyzing the 1p36.13 locus. [file 13015_2023_244_MOESM1_ESM.pdf]

Additional file 1

Note N1: Methods for the Simulation Experiments

The simulation experiments were carried out with the help of a new tool developed specifically for it. It generates a single *seed* haplotype, which then serves to construct a variation graph, on which random walks from source to sink are made to generate new haplotypes. The seed haplotype is initially a sequence of unique markers. A rate of duplications determines the number of duplications to add. For each duplication, the marker to duplicate and the position of insertion are sampled at random. The orientation of the duplicates is sampled according to a ratio of inversions. Next, the seed's *variation graph* is built based on its sequence, represented as a walk through the graph. Finally, a given number of unique haplotypes is generated by performing random walks from source to sink in the graph. Essentially, the simulator starts from seed sequence, then generates an observable set of haplotypes and their graph. Because the walks are random, edges not covered by any of the new haplotypes must be pruned in order to respect the properties of a variation graph. The number of markers and haplotypes, and the ratios of duplication and inversion are the simulation parameters. The ratio of duplications (resp. of inversions) is defined as the ratio of the number of duplications to the number of nodes (resp. number of inversions to the number of duplications). All simulation experiments were carried out by running 50 simulations per parameter set, then applying the solutions of Problems 1 and 3 over the generated graph and haplotype set. The simulation experiments are implemented as *Snakemake* [1] workflows which also provide the benchmarking results then used for evaluation. The data and workflows for the 1p36.13 locus, as well as all simulation experiments are available in the github repository [2] under the `examples` directory.

Note N2: Optimal greedy recombination counting using *generalized suffix arrays*

Here we provide a solution to Problem 2 using a more space-efficient data structure, the *generalized suffix array*. The problem statement remains identical, that is, given a set of terminal sequences $\mathcal{T}$ and a query sequence Q, find a minimal $\mathcal{T}$-block segmentation of Q. Let `pos` be the generalized suffix array of all terminal sequences

$\mathcal{T}$. pos is then the array of indices of all suffixes of the string $S = \mathcal{T} \cup \overline{\mathcal{T}} \cup \$$, sorted in the same lexicographic ordering. In order to achieve the same results as with suffix trees and in linear time, one may compute a *longest common prefix* (lcp) array [3], such that for any suffix at index i, $\texttt{lcp}[i]$ is the maximal value x verifying $S[\texttt{pos}[i-1]..\texttt{pos}[i-1]+x] = S[\texttt{pos}[i]..\texttt{pos}[i]+x]$, with $i \in 2..|\texttt{pos}|$ and $\texttt{lcp}[1] = 0$. lcp can then be used to simulate efficiently a top down traversal of the suffix tree corresponding to pos [4], in linear time and with significantly less space. This relies on *child-intervals*, defined as ranges of suffixes $\texttt{pos}[a..b]$ sharing a longest common prefix of size $\texttt{lcp}[b]$, and in effect child nodes in the would-be suffix tree of S which share the same branching up to $\texttt{lcp}[b]$ characters starting from the root, itself represented by the interval $[1..|\texttt{pos}|]$. The solution is implemented by extending the prefix of a current $\mathcal{T}$-block $Q[i..j]$ with $i \in \{1..|Q|-1\}$, $j \in \{i..|Q|-1\}$, to match that of nested child-intervals $[c..d] \subseteq [a..b]$, with $[a..b] \subseteq [1..|\texttt{pos}|]$, until either $j+1 = |Q|$ and $Q[j+1]$ is a terminal marker, or $Q[i..j+1] \neq S[\texttt{pos}[d]..\texttt{pos}[d]+\texttt{lcp}[d]]$. In the latter case, position j is marked as a recombination, and pattern matching resets at the root against $Q[j..]$. If there is no child-interval $[a..b] \subseteq [1..|\texttt{pos}|]$ such that $Q[j] = S[\texttt{pos}[a]]$, then the solution is infeasible and Q cannot be generated by $\mathcal{T}$. Child-intervals $[c..d] \subseteq [a..b]$ can be computed by performing range minimum queries (RMQs) in $\texttt{lcp}[a+1..b]$. Observe that the minima m in such a range delimit the right boundary d of all child-intervals with a longest common prefix $S[\texttt{pos}[d]..\texttt{pos}[d]+\texttt{lcp}[d]]$, where $\texttt{lcp}[d] > \texttt{lcp}[d+1] > \texttt{lcp}[b]$ and $\texttt{lcp}[d+1] = m$ (unless $d = b$).

Listing 1 summarizes the entire procedure in its simplest form. Listing 2 paraphrases the description in [4] of the supporting GetChild function, which returns the child-interval matching the current character $Q[i]$, with an additional condition to avoid an out-of-bounds access.

Implementation. Simultaneous construction of the generalized suffix array pos and lcp can efficiently be implemented in $O(|S|)$ time using generalizations of induced sorting-based methods[5, 6] suited for constant size alphabets and with a workspace in the order of $|\mathcal{M} \cup \overline{\mathcal{M}}| log|S|$ bits [7]. RMQs can be performed in constant time after appropriate preprocessing in $O(|S|)$ time and workspace [8]. In this algorithm's most naive form, while the size of the alphabet remains constant, GetChild finds child-intervals in $O(|S|)$ time. This can be reduced to $O(log|S|)$ simply by using

Algorithm 1 Suffix array-based recombination counting

```
procedure CountRecSA(Q, S, pos, lcp)
    [l..k] ← [1..|lcp|], i ← 1, r ← 0, z ← 0
    while i ≤ |Q| do
        [a..b] ← GetChild(S, Q[i], pos, lcp, l, k)        ▷ find matching child-interval
        if [a..b] ≠ ∅ then
            if b − a = 1 then                              ▷ match is a leaf node
                n ← |Q| − z                                ▷ must match remainder of Q
            else
                x ← RMQ(lcp[a + 1..b])                     ▷ common prefix length
                n ← min(lcp[x], |Q| − z)                   ▷ do not overshoot Q (shouldn't happen)
            end if
            j ← i + n
            [l..k] = [a..b]                                ▷ update current interval
            while i < j do                                 ▷ match n characters from Q[i]
                if Q[i] ≠ S[pos[l] + i − z] then
                    break
                end if
                i ← i + 1
            end while
        end if
        if [a..b] = ∅ or i ≠ j then                        ▷ no matching child-interval or mismatch above
            if [l..k] = [1..|lcp|] then                    ▷ no further match is possible
                return ∞
            end if
            r ← r + 1                                      ▷ increase recombinations counter
            [l..k] ← [1..|lcp|]                            ▷ reset to top
            i ← i − 1                                      ▷ find interval with last matching character first
            z ← i                                          ▷ save matched characters offset
        end if
    end while
    return r
end procedure
```

binary searches with the already sorted `pos` array. In the extreme case, there can be up to $O(|Q|)$ recombinations, requiring $O(|Q|)$ child queries against the entire `lcp` array. With binary search, the over all time complexity for the counting algorithm is $O(2|S| + |Q|log|S|) = O(|S| + |Q|log|S|)$. Complexity can be improved to $O(|S| + |Q|)$ even without resorting to more efficient data structures such as FM-indices by implementing child and suffix link tables as in [3] or by other simple tricks, such as keeping track of visited sub-intervals in an auxiliary trie built on-line while traversing, to enable fast re-evaluation without much additional space.

Algorithm 2 Fischer and Heun's matching child range query

procedure $\textsc{GetChild}(S, c, \texttt{pos}, \texttt{lcp}, l, r)$
    $p \leftarrow r$
    $r \leftarrow \mathrm{RMQ}(\texttt{lcp}[l+1..p])$ ▷ right bound of first child
    $m \leftarrow \texttt{lcp}[r]$ ▷ minimum across entire interval
    repeat
        if $S[\texttt{pos}[l] + m] = c$ **then**
            return $[l..r-1]$ ▷ found matching child-interval
        end if
        $l \leftarrow r$ ▷ move to next child-interval
        if $l = p$ **then**
            break
        end if
        $r \leftarrow \mathrm{RMQ}(\texttt{lcp}[l+1..p])$
    until $\texttt{lcp}[r] > m$ ▷ query until past last minimum
    if $S[\texttt{pos}[l] + m] = c$ **then** ▷ last interval matches
        return $[l..p]$
    end if
    return $\emptyset$ ▷ queried range cannot contain c
end procedure

References

1. Mölder F, Jablonski KP, Letcher B, Hall MB, Tomkins-Tinch CH, Sochat V, et al. Sustainable data analysis with Snakemake. F1000Research. 2021;10.
2. Bonnet K, Doerr D. Analysis of the set of founder sequences under a homologous recombination model; 2023. https://github.com/marschall-lab/hrfs.
3. Abouelhoda MI, Kurtz S, Ohlebusch E. Replacing suffix trees with enhanced suffix arrays. Journal of Discrete Algorithms. 2004;2(1):53–86. The 9th International Symposium on String Processing and Information Retrieval. Available from: https://www.sciencedirect.com/science/article/pii/S1570866703000650.
4. Fischer J, Heun V. A New Succinct Representation of RMQ-Information and Improvements in the Enhanced Suffix Array. In: Chen B, Paterson M, Zhang G, editors. Combinatorics, Algorithms, Probabilistic and Experimental Methodologies. Berlin, Heidelberg: Springer Berlin Heidelberg; 2007. p. 459–470.
5. Ko P, Aluru S. Space efficient linear time construction of suffix arrays. Journal of Discrete Algorithms. 2005;3(2):143–156. Combinatorial Pattern Matching (CPM) Special Issue. Available from: https://www.sciencedirect.com/science/article/pii/S1570866704000498.
6. Nong G. Practical Linear-Time O(1)-Workspace Suffix Sorting for Constant Alphabets. ACM Trans Inf Syst. 2013 aug;31(3). Available from: https://doi.org/10.1145/2493175.2493180.
7. Louza FA, Gog S, Telles GP. Inducing enhanced suffix arrays for string collections. Theoretical Computer Science. 2017;678:22–39. Available from: https://www.sciencedirect.com/science/article/pii/S0304397517302621.
8. Bender MA, Farach-Colton M. The LCA Problem Revisited. In: Gonnet GH, Viola A, editors. LATIN 2000: Theoretical Informatics. Berlin, Heidelberg: Springer Berlin Heidelberg; 2000. p. 88–94.

Figures

In the following figures, for each of the simulation experiments, performance is measured with regards to a range of values of a single parameter. All others are fixed to a constant value indicated above the given plot. They are labeled as follows: `Nm`, number of markers; `Nh`, number of haplotypes; `Rd`, ratio of duplications; and `Ri`, ratio of inverted duplications. Runtime is measured in seconds of wall clock time, and peak memory usage as the peak PSS in MB.

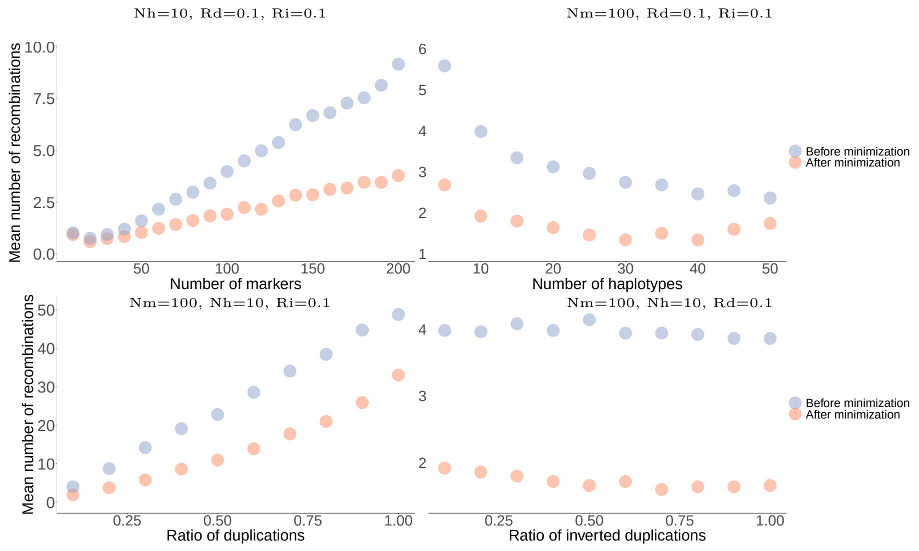

Figure S1 *Reduction in the number of recombinations following minimization.*
The plots show the total number of recombinations before (blue dots) and after (red dots) minimization, as a function of each simulation parameter.

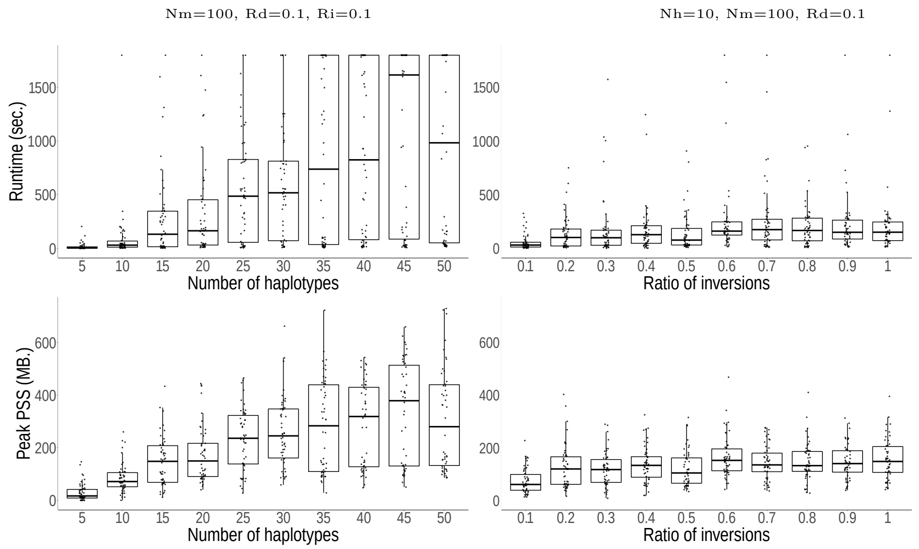

Figure S2 *Number of recombinations minimization benchmarks.*
Runtime (upper panels) and peak PSS (lower panels) as a function of the number of haplotypes (left) and the ratio of inverted duplications (right).

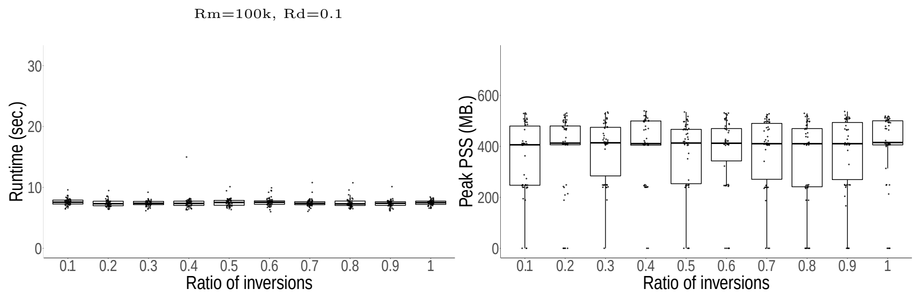

Figure S3 *Flow computation performance with a variable ratio of inversions.*
Runtime (left) and memory usage (right) as a function of this parameter.

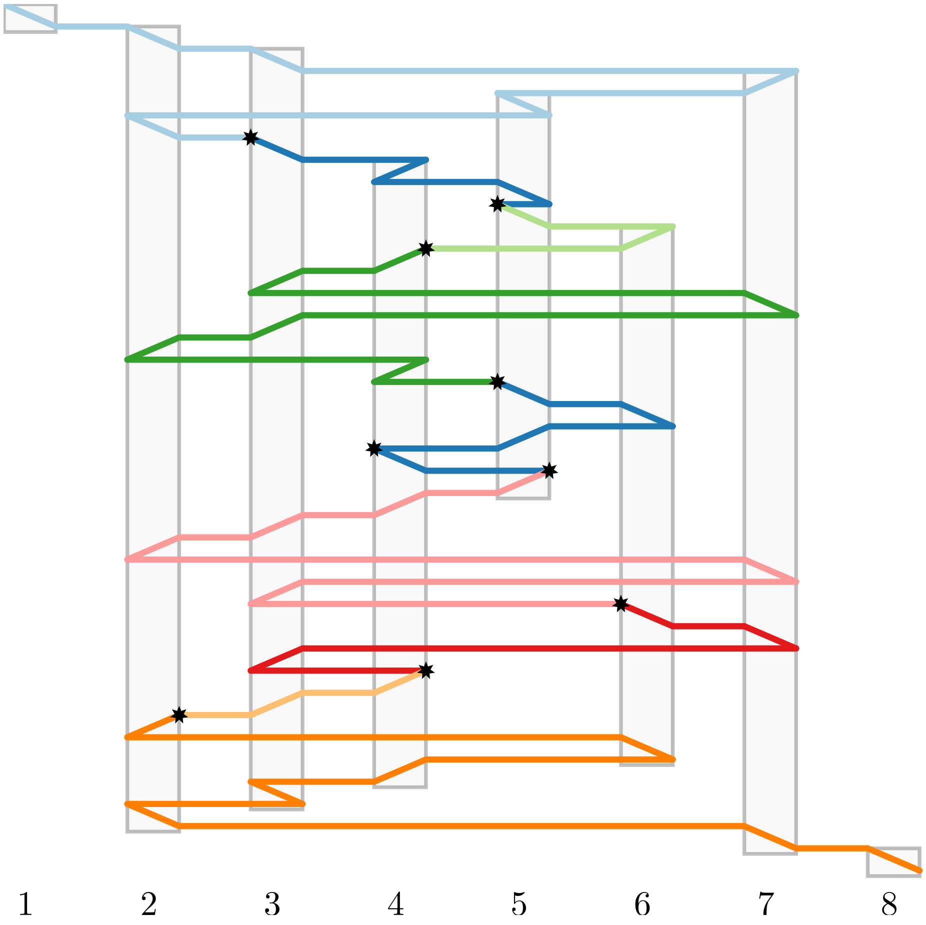

Figure S4 *Visualization of a solution to the minimization problem on the 1p36.13 locus.* The gray bars correspond to the graph's nodes, labeled 1 to 8. The founder sequence (>1>2>3<7>5>2>3<4>5>5<6<4<3>7<3<2 <4>5>6<5>4<5<4<3 <2>7<3>6>7<3<4<3<2>6<4>3>2>7>8) is traced from top to bottom. A slanted line indicates the underlying node being traversed; if slanted rightwards, traversal is in forward direction, and if slanted leftwards, traversal is in reverse direction. Colors correspond to different haplotypes. The haplotype sequence is: *EUR-HG00171-h2, AFR-NA19036-h1, SAS-GM20847-h2, AFR-HG03065-h2, AFR-NA19036-h1, AFR-NA19036-h1, AMR-HG01573-h2, AFR-HG02011-h2, AFR-HG03371-h2, SAS-HG03683-h2.* Recombinations are marked with a star.

Table T1 Sorted haplotype marker sequences used for analyzing the 1p36.13 locus.

Haplotype	Oriented marker sequence
CHM13	>1>2>3>4>2>3>4>5>6<5<4<3<2>7>8
AFR-HG02011-h1	>1>2>3>4>2>3>4<6<5<4<3<2<4<3<2>7>8
AFR-HG02011-h2	>1>2>3<7<6>3>4>5<6<5<4<3<2<4<3<2>7>8
AFR-HG02587-h1	>1>2>3>4>2>3>4<6<4<3<2>6<5<4<3<2<4<3<2>7>8
AFR-HG02587-h2	>1>2>3>4>2>3<7<6>2>3>4>5<6<5<4<3<2>7>8
AFR-HG03065-h1	>1>2>3>4>5<6>3>4>5>6<4<3<2<4<3>6<4<3<2>7>8
AFR-HG03065-h2	>1>2>3>4>2>3<7>3>4>6<4<3<2<4<3>6<4<3<2>7>8
AFR-HG03371-h1	>1>2>3>4>2>3>4<6>3>4>5<6<4<3<2>6<4<3<2<4<3<2>7>8
AFR-HG03371-h2	>1>2>3>4>2>3<7>3>4>5>6<5<4<3<4<3<2>7>8
AFR-NA19036-h1	>1>2>3<4>5>6<5<4<3<2>7<3<2<4<3<2>7>8
AFR-NA19036-h2	>1>2>3<7>3>4>5>6<4<3<2<4<3>6<4<3<2>7>8
AFR-NA19238-h1	>1>2>3>4>2>3>4<6<4<3<2>6<5<4<3<2<4<3<2>7>8
AFR-NA19238-h2	>1>2>3>4>5<6>2>3>4>5<6<5<4<3<2<4<3<2>7>8
AFR-NA19239-h1	>1>2>3<7>2>3>4>5>6<5<4<3<2>7>8
AFR-NA19239-h2	>1>2>3>4>2>3>4>2>3<7<6>2>3>4>5<6<5<4<3<2<4<3<2>7>8
AFR-NA19240-h1	>1>2>3>4>5<6>2>3>4>5<6<5<4<3<2<4<3<2>7>8
AFR-NA19240-h2	>1>2>3<7>2>3>4>5>6<5<4<3<2>7>8
AFR-NA19983-h1	>1>2>3>4>2>3>4>5>6<5<4<3<2>7<3<2>7>8
AFR-NA19983-h2	>1>2>3>4>2>3>4<6>2>3>4>5<6>2>3>4>6<5<4<3<2<4<3<2>7>8
AMR-GM19650-h1	>1>2>3>4>5<6<5<4<3<2<4<3<2>7<3<2<4<3<2>7>8
AMR-GM19650-h2	>1>2>3>4>2>3>4>5>6<5<4<3<2>7<3<2<4<3<2>7>8
AMR-HG00731-h1	>1>2>3<7>2>3>4>5>6<5<4<3<2>7>8
AMR-HG00731-h2	>1>2>3<7>2>3>4>5<6<5<4<3<2>6<4<3<2>7>8
AMR-HG00732-h1	>1>2>3>4>5>6<4<3<2>6<5<4<3<2>7>8
AMR-HG00732-h2	>1>2>3>4>2>3>4>2>3>4>5>6<5<4<3<2<4<3<2>7>8
AMR-HG00733-h1	>1>2>3<7>2>3>4>5>6<5<4<3<2>7>8
AMR-HG00733-h2	>1>2>3>4>5>6<4<3<2>6<5<4<3<2>7>8
AMR-HG01114-h1	>1>2>3<7>2>3>4>5>6<5<4<3<2>6<4<3<2>7>8
AMR-HG01114-h2	>1>2>3>4>2>3>4>5<6<5<4<3<2>6<5<4<3<2>7>8
AMR-HG01573-h1	>1>2>3>4>5>6<5<4<3<2>6<5<4<3<2<4<3<2>7>8
AMR-HG01573-h2	>1>2>3<7>2>3>4>5<4<3<2<5<4<3<2>7>8
EAS-GM00864-h1	>1>2>3<7>2>3>4>5>6<5<4<3<2>6<4<3<2>7>8
EAS-GM00864-h2	>1>2>3>4>2>3>4>5>6<5<5<5<4<3<2>7>8
EAS-GM18939-h1	>1>2>3<7>2>3>4>5>6<5<4<3<2>6<4<3<2>7>8
EAS-GM18939-h2	>1>2>3<7>2>3>4>6<5<4<3<2>6<4<3<2>7>8
EAS-HG00512-h1	>1>2>3<7>2>3>4>5>6<5<4<3<2>6<4<3<2>7>8
EAS-HG00512-h2	>1>2>3>4>5<6>2>3>4>5>6<5<4<3<2>6<4>3>2>7>8
EAS-HG00513-h1	>1>2>3<7>2>3>4>5<6>2>3>4>5>6<5<4<3<2>6<4<3<2>7>8
EAS-HG00513-h2	>1>2>3<7>2>3>4>5<6>2>3>4>5>6<5<4<3<2>6<4<3<2>7>8
EAS-HG00514-h1	>1>2>3>4>5<6>2>3>4>5>6<5<4<3<2>6<4>3>2>7>8
EAS-HG00514-h2	>1>2>3<7>2>3>4>5>2>3>4>5>6<5<4<3<2>6<4<3<2>7>8
EAS-HG01596-h1	>1>2>3<7>2>3>4>5>6<5<4<3<2>7>8
EAS-HG01596-h2	>1>2>3<7>2>3>4>5>6<5<4<3<2>6<4<3<2>7>8
EAS-HG02018-h1	>1>2>3<7>2>3>4>5>6<5<4<3<2>6<4<3<2>7>8
EAS-HG02018-h2	>1>2>3<7>2>3>4>5>6<5<4<3<2>6<4<3<2>7>8
EAS-NA18534-h1	>1>2>3<7>2>3>4>5>6<5<4<3<2>6<4<3<2>7>8
EAS-NA18534-h2	>1>2>3>4>5>2>3>4>5<6<5<4<3<2>6<5<4<3<2>7>8
EUR-GM12329-h1	>1>2>3>4>5>6<5<4<3<2>6<4<3<2>7>8
EUR-GM12329-h2	>1>2>3>4>2>3>4>5>6<5<4<3<2>7>8
EUR-GM20509-h1	>1>2>3>4>5>6<5<4<3<2>6<4<3<2>7>8
EUR-GM20509-h2	>1>2>3<7<6>2>3>4>5>6<5<4<3<2>6>7<3<2<4<3<2>7>8
EUR-HG00096-h1	>1>2>3<7>2>3>4>5<6<5<4<3<2>6<4<3<2>7>8
EUR-HG00096-h2	>1>2>3>4>5>2>3>4<6<5<4<3<2>7>8
EUR-HG00171-h1	>1>2>3>4>2>3>4>5>6<5<4<3<2>6<4<3<2>7>8
EUR-HG00171-h2	>1>2>3<7>5>2>3>4>5>2>3>4>6<5<4<3<2>7>8
EUR-HG01505-h1	>1>2>3<7>2>3>4>5>6<5<4<3<2>6<4<3<2>7>8
EUR-HG01505-h2	>1>2>3>4>2>3>4>5>2>3>4>5<6<5<4<3<2>7>8
EUR-NA12878-h1	>1>2>3>4>2>3>4>5>6<5<4<3<2>6<5<4<3<2<4<3<2>7>8
EUR-NA12878-h2	>1>2>3>4>2>3>4>5>6<5<4<3<2>6<5<4<3<2>7>8
SAS-GM20847-h1	>1>2>3>4>5<6<5<4<3<2>6<5<4<3<2>6<5<4<3<2>7>8
SAS-GM20847-h2	>1>2>3>4>2>3>4>5>6<5<5<4<3<2>6<4<3<2>7>8
SAS-HG02492-h1	>1>2>3>4>2>3>4>5>6<5<4<3<2>6<5<4<3<2<4<3<2>7>8
SAS-HG02492-h2	>1>2>3>4>2>3>4>2>3>4>5>6<4<3<2>6<5<4<3<2<4<3<2>7>8
SAS-HG03009-h1	>1>2>3<7>2>3>4>5>6<5<4<3<2>6<4<3<2>7>8
SAS-HG03009-h2	>1>2>3>4>5>2>3>4>5<6<5<4<3<2>7>8
SAS-HG03683-h1	>1>2>3>4>2>3>4>5>6<5<4<3<2>7>8
SAS-HG03683-h2	>1>2>3>4>5<6>2>3>4>5>6<5<4<3<2>6<4>3>2>7>8
SAS-HG03732-h1	>1>2>3>4>2>3>4>5>6<5<4<3<2>7>8
SAS-HG03732-h2	>1>2>3>4>5>6<5<4<3<2>6<4<3<2>7>8

The sequences are listed in alphabetical order and in GFA Path format, where > corresponds to traversal in forward direction, and < in reverse direction. The haplotype labeled CHM13 is the provided reference.
